# Proteomic analysis enables distinction of early‐ versus advanced‐stage lung adenocarcinomas

**DOI:** 10.1002/ctm2.106

**Published:** 2020-06-14

**Authors:** Olga Kelemen, Indira Pla, Aniel Sanchez, Melinda Rezeli, Attila Marcell Szasz, Johan Malm, Viktoria Laszlo, Ho Jeong Kwon, Balazs Dome, Gyorgy Marko‐Varga

**Affiliations:** ^1^ Clinical Protein Science and Imaging, Biomedical Center Department of Biomedical Engineering Lund University Lund Sweden; ^2^ Department of Translational Medicine Lund University Malmö Sweden; ^3^ Cancer Center Semmelweis University Budapest Hungary; ^4^ Department of Surgery Division of Thoracic Surgery Comprehensive Cancer Center Medical University of Vienna Vienna Austria; ^5^ Chemical Genomics Global Research Lab Department of Biotechnology College of Life Science and Biotechnology Yonsei University Seoul Republic of Korea; ^6^ Department of Tumor Biology National Korányi Institute of Pulmonology Budapest Hungary; ^7^ Department of Thoracic Surgery Semmelweis University and National Institute of Oncology Budapest Hungary

**Keywords:** clinical proteomics, lung adenocarcinomas, mass spectrometry, proteomics

## Abstract

**Background:**

A gel‐free proteomic approach was utilized to perform in‐depth tissue protein profiling of lung adenocarcinoma (ADC) and normal lung tissues from early and advanced stages of the disease. The long‐term goal of this study is to generate a large‐scale, label‐free proteomics dataset from histologically well‐classified lung ADC that can be used to increase further our understanding of disease progression and aid in identifying novel biomarkers.

**Methods and results:**

Cases of early‐stage (I‐II) and advanced‐stage (III‐IV) lung ADCs were selected and paired with normal lung tissues from 22 patients. The histologically and clinically stratified human primary lung ADCs were analyzed by liquid chromatography‐tandem mass spectrometry. From the analysis of ADC and normal specimens, 4863 protein groups were identified. To examine the protein expression profile of ADC, a peak area‐based quantitation method was used. In early‐ and advanced‐stage ADC, 365 and 366 proteins were differentially expressed, respectively, between normal and tumor tissues (adjusted *P*‐value < .01, fold change ≥ 4). A total of 155 proteins were dysregulated between early‐ and advanced‐stage ADCs and 18 were suggested as early‐specific stage ADC. In silico functional analysis of the upregulated proteins in both tumor groups revealed that most of the enriched pathways are involved in mRNA metabolism. Furthermore, the most overrepresented pathways in the proteins that were unique to ADC are related to mRNA metabolic processes.

**Conclusions:**

Further analysis of these data may provide an insight into the molecular pathways involved in disease etiology and may lead to the identification of biomarker candidates and potential targets for therapy. Our study provides potential diagnostic biomarkers for lung ADC and novel stage‐specific drug targets for rational intervention.

## 1 INTRODUCTION

Lung cancer is today one of the leading causes of cancer deaths worldwide. Despite a poor 5‐year survival rate of 15%, no improvement in survival has occurred for decades.^1^ Nonsmall cell lung cancer (NSCLC) is subdivided into three major histological types: squamous cell carcinoma, large cell carcinoma, and adenocarcinoma (ADC). ADC of the lung is the predominant histological type of lung cancer and accounts for 40% of all NSCLC cases. Although lung ADC is associated with smoking, it is the most frequent lung malignancy in individuals who have never smoked.^2^

Traditionally, therapeutic strategies for NSCLC are based on tumor histology and stage. At the early stage, surgical resection is the most effective treatment. The standard first‐line therapy for patients with inoperable, advanced NSCLC has been to employ different chemotherapy modalities. Although specific molecular‐targeted therapies for the treatment of distinct subtypes of NSCLC have been developed, treatment options for the majority of patients remain unsatisfactory. Therefore, new treatment strategies with greater efficacy and lower toxicity are essential. Additionally, both diagnostic and prognostic biomarkers are urgently required to increase patient survival.

Recent advancements in high‐throughput molecular biology technologies have deepened our understanding of the pathology underlying NSCLC and highlighted the significant heterogeneity of NSCLC.^3^ Sequencing of entire cancer genomes has resulted in the identification of driver mutations and frequently altered signaling pathways. This approach has led to the definition of new molecular subtypes of NSCLC (EGFR, ALK, TP53, KRAS, and ROS1) and new treatment options.^4^, ^5^, ^6^, ^7^

The prognosis for lung cancer patients is strongly related to the stage of the disease at the time of diagnosis. Patients with localized disease have a 5‐year survival rate of 52%, meanwhile, patients diagnosed with the distal disease have a dismal 5‐year survival rate of 3.6%.^2^ Only one third of lung cancer cases, however, are diagnosed at an early stage.^8^ Therefore, early diagnosis plays a crucial role in reducing lung cancer mortality. The current screening methods mainly include the histopathological examination of biopsies, lung imaging, and biochemical screens for several specific biomarkers. Several potential biomarkers have already been identified. These include the genes CEA, CYFRA21‐1, KRAS, and TP53^9^; however, the majority of these biomarkers fail to show a strong specificity and sensitivity for early‐stage lung cancer.

Proteins are implicated in all biological processes; thus, these play an essential role in disease progression. Therefore, large‐scale and systematic analyses of proteins have become an important tool for tumor characterization. Proteomic methods based on mass spectrometry (MS) have emerged as powerful tools to discover diagnostic, prognostic, and therapeutic protein biomarkers.^10^ Various MS‐based approaches have been used to identify differentially expressed proteins (DEPs) in lung ADC cells, tissues, and biological fluids.^11^ Due to the low cost, minimal sample handling and manipulation, and high throughput, the application of label‐free proteomics to investigate differential protein expression of clinical samples has gained considerable attention.

Fresh tissue is difficult to collect for clinical proteomic studies; therefore, FFPE (formalin‐fixed and paraffin‐embedded) tissues are the most frequently used and are easily preserved for subsequent clinical diagnoses. FFPE tissues are routinely collected for clinical diagnosis; however, due to the presence of formalin‐induced protein crosslinks and modifications,^12^ protein recovery from FFPE tissues is difficult. Proteomic analysis of fresh frozen tissues may reflect more accurately the in vivo tissue proteome. Therefore, surgically resected fresh frozen tissues were used in this study.

Label‐free, gel‐free proteomics has been widely used to describe large‐scale biological systems.^13^ Label‐free MS‐based proteomic methods provide relative protein abundance in normal and cancer tissues that may aid in obtaining a deeper insight into molecular interactions, signaling pathways, and biomarker identification. In the current study, a proteomic analysis was conducted using a label‐free liquid chromatography (LC)‐MS approach to systematically assess the stage‐specific signaling pathways and potential markers. This enabled high‐throughput, semiquantitative assessment of protein abundances in a complex mixture. These results provide a deeper insight into the proteomes of early‐ and advanced‐stage lung ADC.

## 2 MATERIALS AND METHODS

### 2.1 Sample selection

We collected malignant and adjacent nonmalignant lung tissue samples from 22 patients between 46 and 74 years old (median: 60 years) with ADC histology. Out of 22, 11 patients were in the early‐stage of ADC (pathological stage I and II disease) and 11 had advanced‐stage ADC (pathological stage III and IV disease) (Table 1). Malignant and adjacent nonmalignant lung tissue samples were harvested in the operating theater from patients undergoing resection or lobectomy for NSCLC. The matched control lung tissue was excised from regions that were distant (8‐10 cm) from the tumor. All procedures were approved by institutional IRB (2521‐0/2010‐1018EKU) protocols with informed patient consent. Tissue samples were promptly frozen in liquid nitrogen and stored at –80°C. Specimens were annotated with age, gender, race, diagnosis (including stage), smoking status, and the number of pack years. Criteria for patient selection were as follows: current or former smokers; diagnosis of NSCLC ADC; early stage (I‐II) or advanced stage (III‐IV) (Table 1). Detailed information of each patient and Hematoxylin and Eosin (H&E) images can be accessed in Supplementary File F1.

**TABLE 1:** Patient characteristics

Characteristics	n (%)
Total number of patients	22 (100%)
Sex	
Female	13 (59.1%)
Male	9 (40.9%)
Age at the diagnosis, years	
Median (range)	60 (46‐74)
<60	9 (40.9%)
≥60	13 (59.1%)
Smoker	
Current smoker	10 (45.5%)
Former smoker	11 (50 %)
Unknown	1 (4.5%)
T stage	
T1	5 (22.7%)
T2	13 (59.1%)
T3	4 (18.2%)
N stage	
N0	10 (45.5%)
N1	4 (18.2%)
N2	8 (36.3%)
Disease stage	
Ia (early)	4 (18.2%)
I1a (early)	4 (18.2%)
I1b (early)	3 (13.6%)
IIIa (advanced)	9 (40.9%)
IV (advanced)	2 (9.1%)

### 2.2 Sample preparation

Preparation of tissues followed by protein extraction using buffer exchange was performed as previously described.^14^ In brief, frozen tissue samples from each tumor were sliced into 10 × 10 μm sections using a cryotome. Tissue sections were then homogenized in lysis buffer (50 mM ammonium bicarbonate, 6 M urea) and incubated for 30 min on ice. Samples were sonicated, clarified by centrifugation for 10 min (10 000 × *g*, 4°C), and the supernatant transferred to a clean microcentrifuge tube. Total protein concentration was determined using the BCA protein assay kit (Pierce, Thermo Fischer Scientific). Proteins were reduced with 10 mM Dithiothreitol (DTT) (1 h at 37°C) and alkylated using 40 mM iodoacetamide (30 min, in the dark at room temperature) followed by buffer exchange with 50 mM ammonium bicarbonate buffer (pH 7.6). A total of 50 μg total protein was digested overnight at 37°C with trypsin at an enzyme to protein ration of 1:50 w/w. The digested peptides were concentrated and desalted with C18 MicroSpin columns, and lyophilized and resuspended in 0.1% formic acid +5 fmol/μL PRTC (Pierce Peptide Retention Time Calibration Mixture).

**Graphical Headlights**

1. Mass spectrometry analysis revealed changes at the protein level between early‐ and advanced‐stage lung adenocarcinomas.
2. Most of the protein‐enriched pathways overexpressed in tumor ADCs are involved in mRNA metabolism.
3. The proteomics analysis identified 18 early‐specific proteins.
4. Potential diagnostic biomarkers were identified for lung ADC and novel stage‐specific drug targets for rational intervention.

### 2.3 Proteomic analysis and database searching

Samples (peptides produced by digestion) were analyzed by triplicate in a randomized order using a Q‐Exactive Plus mass spectrometer connected to an Easy‐nLC 1000 pump (Thermo Scientific, San José, CA) with a top 10 DDA method (2 μL, 1 μg on the column). Peptides were loaded onto an Acclaim PepMap 100 precolumn (75 μm × 2 cm, Thermo Scientific), and separated on an easy‐Spray column (25 cm × 75 μm ID, PepMap C18 2 μm, 100 Å) with the flow rate set to 300 nL/min and the column temperature to 35°C. A nonlinear 90 min gradient was applied, using solvent A (0.1% formic acid) and solvent B (0.1% formic acid in acetonitrile). Full MS scans were acquired with the Orbitrap mass analyzer over *m*/*z* 400‐1600 range and the Target Automated Gain Control (AGC) value was set to 1 × 10^6^ and maximum injection time of 100 ms. The 10 most intense peaks with charge state ≥2 were fragmented in the Higher‐energy Collisional Dissociation (HCD) collision cell with a normalized collision energy of 26%. Tandem mass spectra were acquired in the Orbitrap mass analyzer with a resolution of 17 500 (at *m*/*z* 200), target AGC value of 5 × 10^4^ and maximum injection time of 100 ms. The underfill ratio was set to 10% and dynamic exclusion was 45 s.

Raw files were analyzed with Proteome Discoverer v2.1 (Thermo Scientific). Proteins were searched against the UniProtKB human database using the SEQUEST HT search algorithm that is integrated into Proteome Discoverer. The search was performed with the following parameters: carbamidomethylation of cysteine residues and oxidation of methionine residues as static and dynamic modifications, respectively; and mass tolerances of 10 ppm for precursor ion and 0.02 Da for fragment ions. Up to two missed cleavages for tryptic peptides were allowed. The filters “high confidence” and “at least two unique peptides per protein” were also applied (false discovery rate [FDR] < .01).

Peptide and protein quantitation was assessed using the converted mzxml files^15^ (MSconvert) and analyzed by OpenMS v.2.0.0 and TOPP^16^ using X‐tandem as search engine against the UniProt human database (Human, 9606; reviewed, 20 165). The search included carbamidomethylation of cysteine residues and oxidation of methionine residues as static and dynamic modifications, respectively. FDR was determined by searching a reversed database and was set to < .01 for proteins and peptides (at least two unique peptides/protein). Enzyme specificity was “trypsin” and ‘two miscleavages’ were permitted with a minimum of seven amino acids per identified peptide. Peptide identification was based on a search with an initial mass deviation for the precursor and fragment ions of up to 10 ppm and 0.02 Da, respectively. To match peptide identifications across different replicates and adjacent samples by condition, a match‐between‐runs was performed.

For the quality control (QC) of the LC‐MS analysis, four of the most intense peaks observed in all LC‐MS runs of previous analyses performed in our laboratory with lung tissues samples were selected as QC reference (see Table F1‐S1 of the File F2 in the Supporting Information). In addition, mass error distribution for peptide groups and the distribution of peptide by retention times across the LC‐MS is showed. See the results of the analysis in File F2 in the Supporting Information.

### 2.4 Bioinformatics

Statistical analyses and data visualization were performed in Perseus^17^ (v1.6.0.2) and R.^18^, ^19^ In order to obtain >70% valid values per protein in at least one condition, data were filtered based on missing values. Missing values were replaced using a “data imputation” algorithm to simulate signals of low‐abundance proteins under the assumption that these are biased toward the detection limit of the MS measurement. In Perseus, a width of 0.3 and a downshift of 1.8 were chosen to draw random numbers from a normal distribution. The intensities were normalized by applying a log_2_ transformation and then standardized by subtracting the median. An overall picture of the proteomics results was assessed by performing a principal component analysis (PCA)^20^, ^21^ based on the expression of all proteins quantified in all samples. To determine DEPs, an ANOVA test was initially performed to detect changes across the four sample groups. To compare normal and tumor tissues within the disease stages, a paired Student *t*‐test (two‐tailed) was then performed; and to compare tissues of early versus advanced stages, an unpaired Student *t*‐test (two‐tailed) was applied. In all cases, *P*‐values were adjusted to obtain a FDR < 1%. Proteins with *q*‐values < .01 and fold change (FC) ≥ 4 were considered differentially expressed. To visualize the behavior of DEPs across the time points, unsupervised hierarchical clustering (distance: “euclidean"; linkage method: “complete”) was performed. The Spearman rank test was performed to analyze the coefficient of correlation between selected DEPs. Correlations with an *P*‐value < .05 and *r* > .5 were considered significant. Gene ontology (GO) and Reactome pathway enrichment analyses were performed using the bioinformatics web tool PANTHER (http://www.pantherdb.org/).^22^ This tool was also used to perform overrepresentation test (*P* < .05) where all identified proteins were used as the background list. STRING database was used to assess functional protein association networks (https://string-db.org/).

## 3 RESULTS

### 3.1 Lung ADC tissue proteomics

**FIGURE 1:**
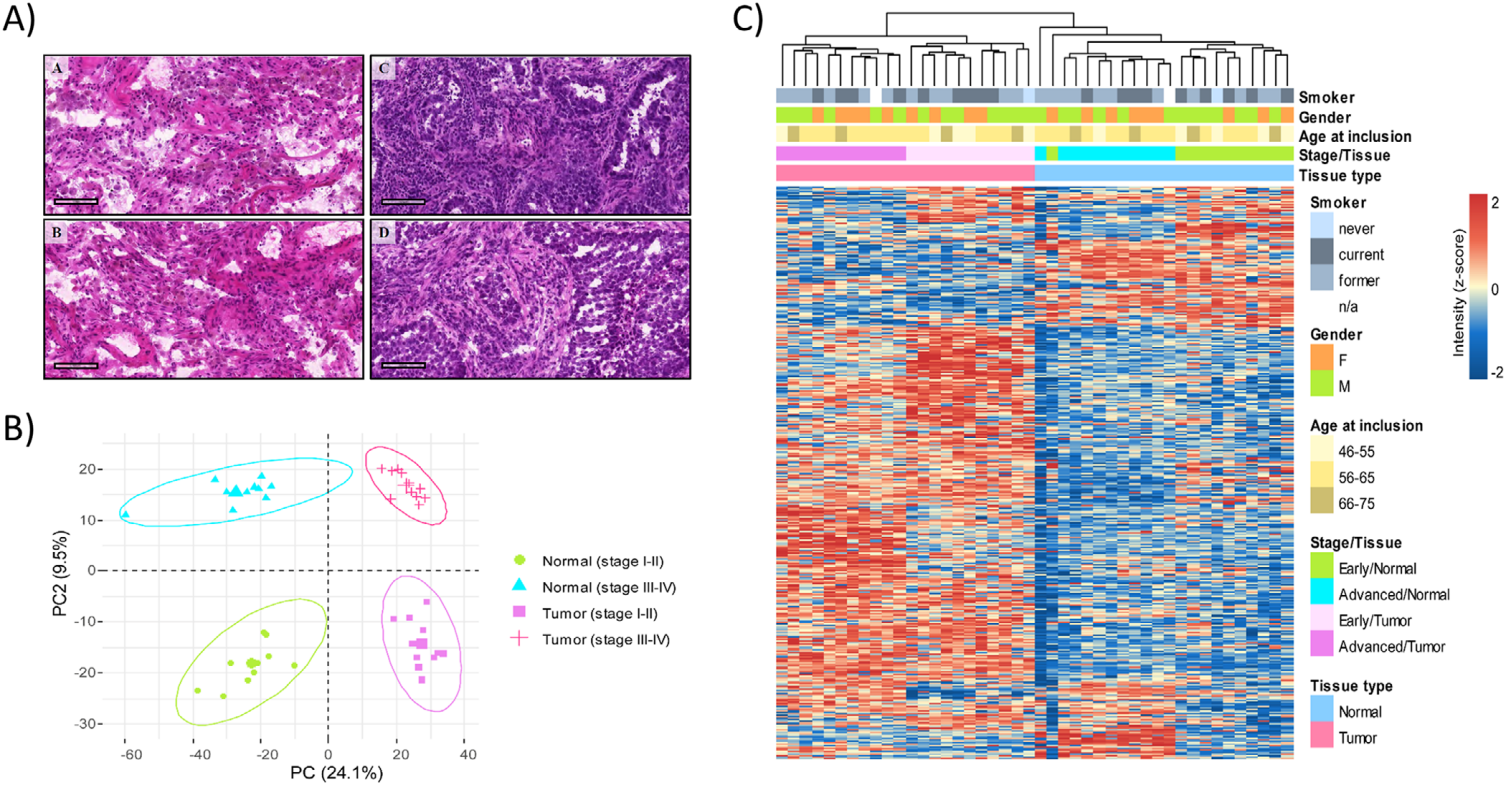
**A,** Representative H&E staining of tumor and normal adjacent tissue samples. It illustrates the representative tumor (right) and normal adjacent (left) tissue sections from early‐stage ADC (A) and (C), and from advanced‐stage ADC (B) and (D). **B,** Principal component analysis (PCA) of protein expression patterns of the 44 samples. The greatest variance (PC1_24.1%) is given by the differences between normal and tumor tissue and the second (PC2_ 9.5%) by differences between stages. **C,** Hierarchical clustering separated normal, early, and advanced ADC proteomes. Heat map and hierarchical clustering based on 1579 differentially expressed proteins (DEPs) from the ANOVA test. Heat map colors are based on the *z*‐scored (log_2_) intensity values. Blue and red correspond to decreased and increased expression levels, respectively

In this study, differences at the protein level between early‐stage and advanced‐stage lung ADC tissues were assessed. Malignant tumor samples and their nonmalignant adjacent tissue were also compared separately for early‐ and advanced‐stage ADC. As shown in Figure 1A, the histology of the tissue samples was confirmed (Hematoxylin and Eosin [H&E] stained sections of tumor and adjacent normal lung tissue samples can be found in File F1 in the Supporting Information). Following enzymatic digestion, the extracted protein samples were individually analyzed on a Q‐Exactive Plus Orbitrap coupled to peptide separation by LC.

Proteomic profiling was performed on matched malignant and normal tissues and a total of 4863 proteins were identified across all 22 tissue pairs (44 samples) (see Table S1). Using PCA, based on the protein expression of all proteins quantified in all samples, a 2D scatterplot was generated to explore and distinguish all analyzed groups. In Figure 1B, the first versus the second principal component (PC) are represented. Importantly, the first PC (24.1% of explained variances) clearly separated control (“normal”) tissues (left) from tumor tissues (right). When focusing on the second PC (9.5% of explained variances), the data showed early‐stage ADC and matched control tissues at the bottom of the plot, whereas advanced ADCs and matched controls are shown at the top. To detect overall changes across the four sample groups, an ANOVA test was performed and 1579 proteins result differentially expressed (FDR < 1%) (see Table S2A). Based on the intensity of the DEPs, unsupervised hierarchical clustering of the 44 datasets confirmed that the normal, early, and advanced ADC proteomes were sufficiently distinct to be resolved from one another (Figure 1C) independently of other clinical characteristics of the patients (e.g., gender, age at inclusion, smoking status).

**FIGURE 2:**
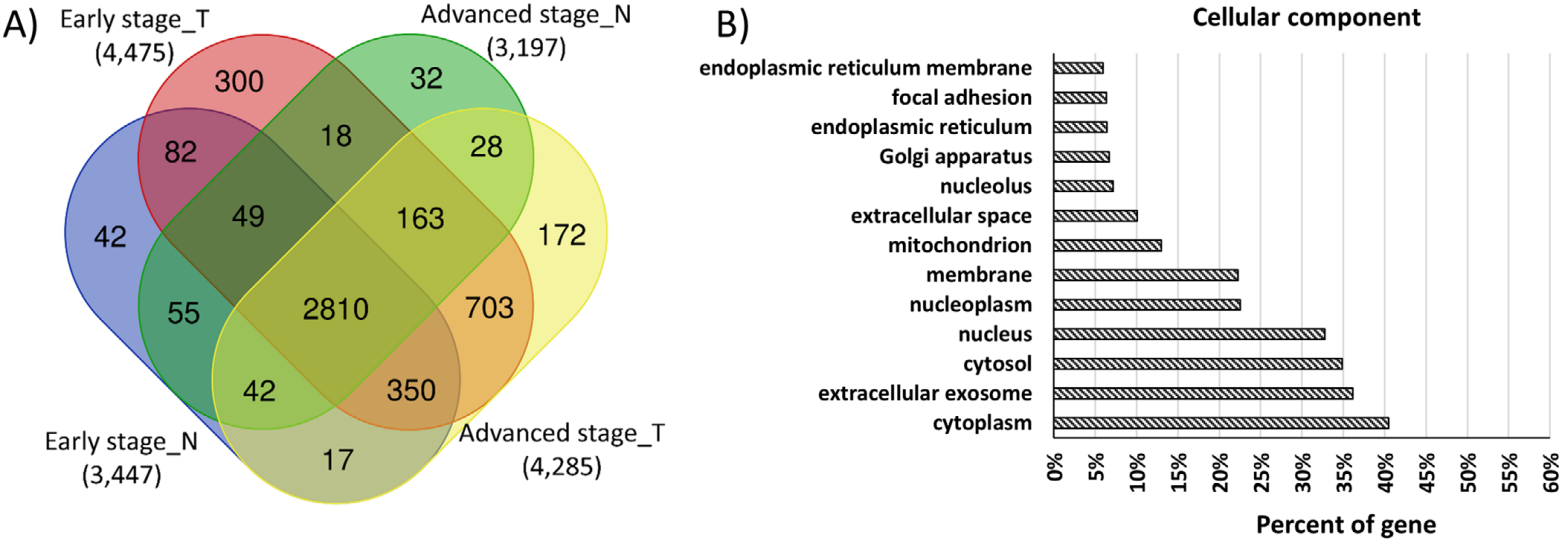
**A,** Venn diagrams showing the distribution of the identified protein groups across the sample classifications. T refers to tumor samples. N refers to adjacent normal tissue sample. **B,** Gene ontology (GO) annotation of identified proteins by cellular component

Our primary goal was to determine the differences in protein expression profiles between tumor and tissues with normal histology. Of the 4863 proteins, 2810 proteins (58%) were observed across all groups, whereas 703 proteins (14.5 %) were shared among the advanced‐ and the early‐stage ADC groups. A total of 300 and 172 proteins were specific to early‐ and advanced‐stage tumors, respectively (Figure 2A). When the number of proteins identified in the ADC tissues was compared to the matched control tissues, an increase in the complexity of the proteome in the tumor tissue was revealed. This observation is similar to the results obtained by Tenzer et al^23^ (average protein number in normal tissue: 3322; average protein number in ADC: 4380). To obtain an overview of the cellular distribution of the identified proteins, these were classified according to the cellular component category of GO. Concerning cellular component, the majority of the proteins were assigned to the cytoplasm (40.5%), whereas 36.1% and 10% mapped to extracellular exosome and space, respectively. A total of 1677 (35%) proteins mapped to the cytosol, 2657 (55,2%) to nucleus and nucleoplasm, whereas 1070 (22.3%) to the membrane (Figure 2B). Overall, the distribution of the proteins was not biased toward a specific cell compartment.

### 3.2 Differential protein expression between normal and tumor samples: Early stage

**FIGURE 3:**
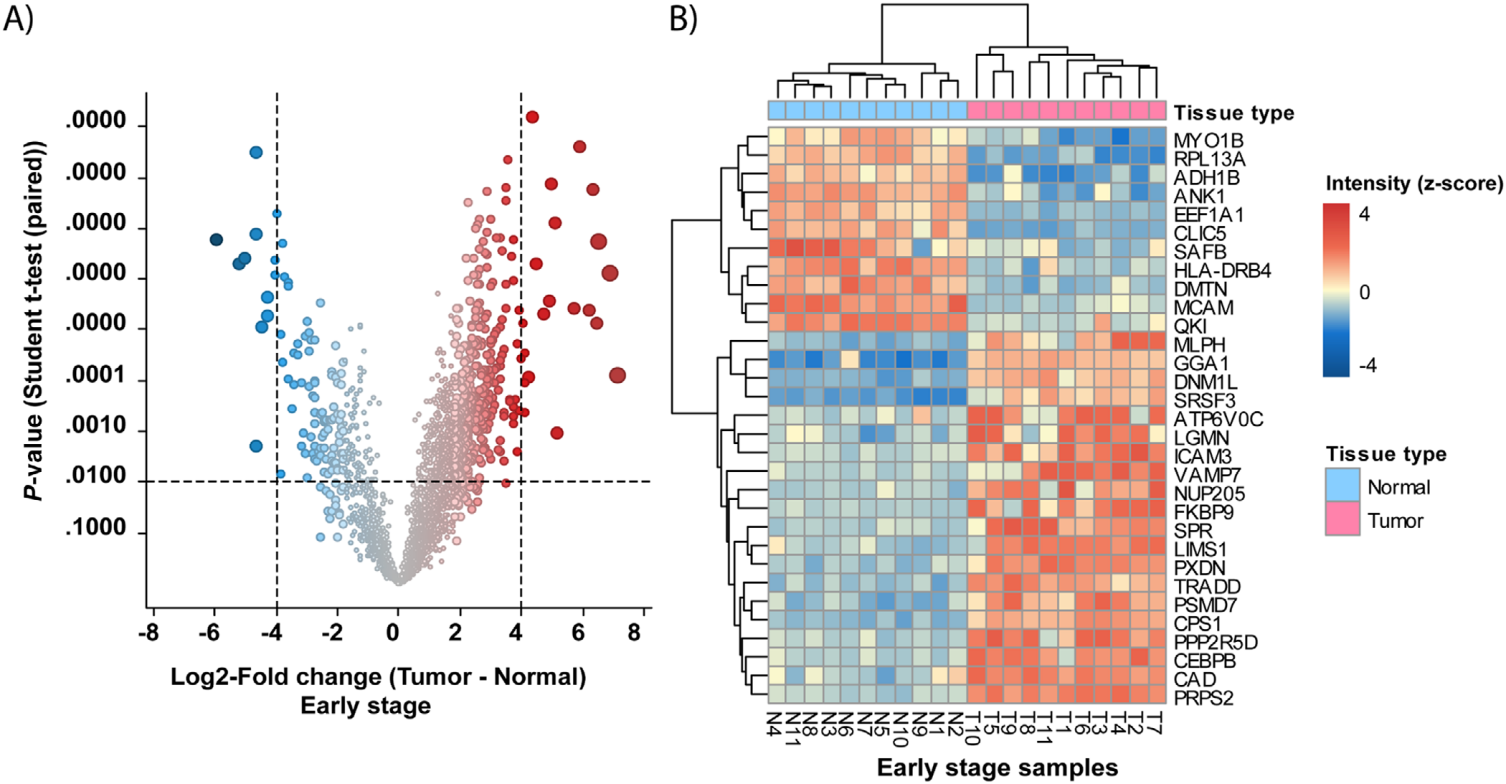
Differentially expressed proteins in early‐stage ADC. **A,** Volcano plot of log_2_‐fold change (FC) versus *P*‐value *t*‐test. Points alter in size according to the magnitude of the fold change. **B,** Heat map generated by hierarchical clustering of the most altered proteins. The heat map shows a clear separation of adjacent normal and tumor tissues (–4 ≥ log_2_ FC ≥ 4, adj. *P* < .01). Blue and red correspond to decreased and increased expression levels, respectively

Comparison of the DEPs (–2 ≥ log_2_ FC ≥ 2; *q*‐value < .01) between normal and tumor tissues showed that in early‐stage tumor, 86 and  279 proteins were down‐ and upregulated, respectively (see Table S2B). The proteins with the greatest alteration in expression (–4 ≥ log_2_ FC ≥ 4) are depicted in Figure 3B as a heat map. The within‐patient comparison between tumor and normal tissue is shown in Figure S1A for each patient with early‐stage ADC.

The pathway analysis revealed that the upregulated proteins in tumor were associated with translation initiation and regulation of translation, such as nonsense‐mediated decay and mRNA splicing (Table S3). Specifically, 12, 9, and 6 proteins were identified as ribosomal proteins, mRNA‐splicing factors, and translation factors, respectively (Table S4).

A total of 224 proteins were exclusively quantified in the early‐stage tumor tissues (in more than 70% of the samples), but not in the matched normal samples. Among these, 67 were identified in all the early‐stage tumor samples (Table S5).

To assess potential pathways associated with early‐stage ADC, these 236 proteins were further analyzed using the bioinformatics tool PANTHER. The GO‐Slim biological process analysis revealed that these proteins are involved in mRNA splicing, DNA replication, and regulation of cell cycle (Table S6).

### 3.3 Differential protein expression between normal and tumor samples: Advanced stage

**FIGURE 4:**
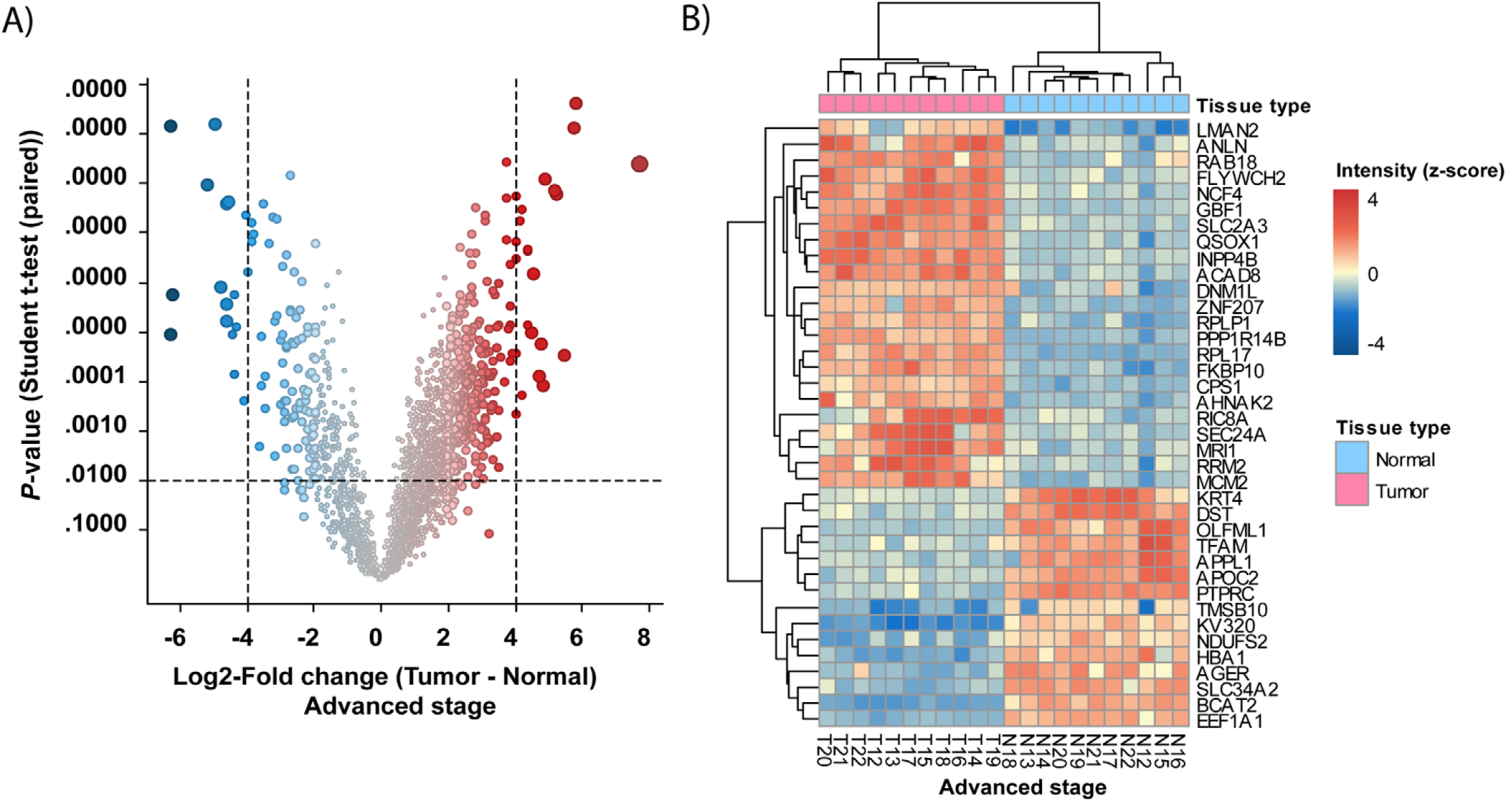
Comparison between normal and tumor tissue in advanced‐stage ADC. **A,** Volcano plot of log_2_‐fold change (FC) versus *P*‐value *t*‐test. Points alter in size according to the magnitude of the fold change. **B,** Heat map generated by hierarchical clustering of the most altered proteins. The heat map shows a clear separation of adjacent normal and tumor tissues (–4 ≥ log_2_ FC ≥ 4, adj. *P* < .01). Blue and red correspond to decreased and increased expression levels, respectively

Comparative analysis of the advanced‐stage tumor tissues showed that 92 and 274 proteins were down‐ and upregulated, respectively, relative to the normal paired samples (–2 ≥ log_2_ FC ≥2; *q*‐value < .01) (see Table S2C). Figure 4 shows a heat map performed with proteins that had the highest expression level alterations (–4 ≥ log_2_ FC ≥ 4). The within‐patient comparison between tumor and normal tissue is shown in Figure S1B for each patient with advanced‐stage ADC.

Pathway enrichment analysis of the 274 upregulated proteins revealed that a variety of cellular processes are overrepresented in advanced‐stage lung ADC (Table S7). The upregulated proteins and enriched pathways are associated with mRNA splicing, translation, and regulation of translation. Specifically, seven proteins belong to the family of mRNA splicing factors, seven ribosomal proteins were identified, and seven proteins are aminoacyl‐tRNA synthetases (ARS) (Table S8). ARSs are important housekeeping proteins that play an essential role in protein synthesis. Accumulating evidence indicates that ARSs play an important role in cancer and it has been demonstrated that some ARSs show cancer‐associated overexpression.^24^, ^25^, ^26^

### 3.4 Proteins differentially‐expressed between early‐ and advanced‐stage ADC

**FIGURE 5:**
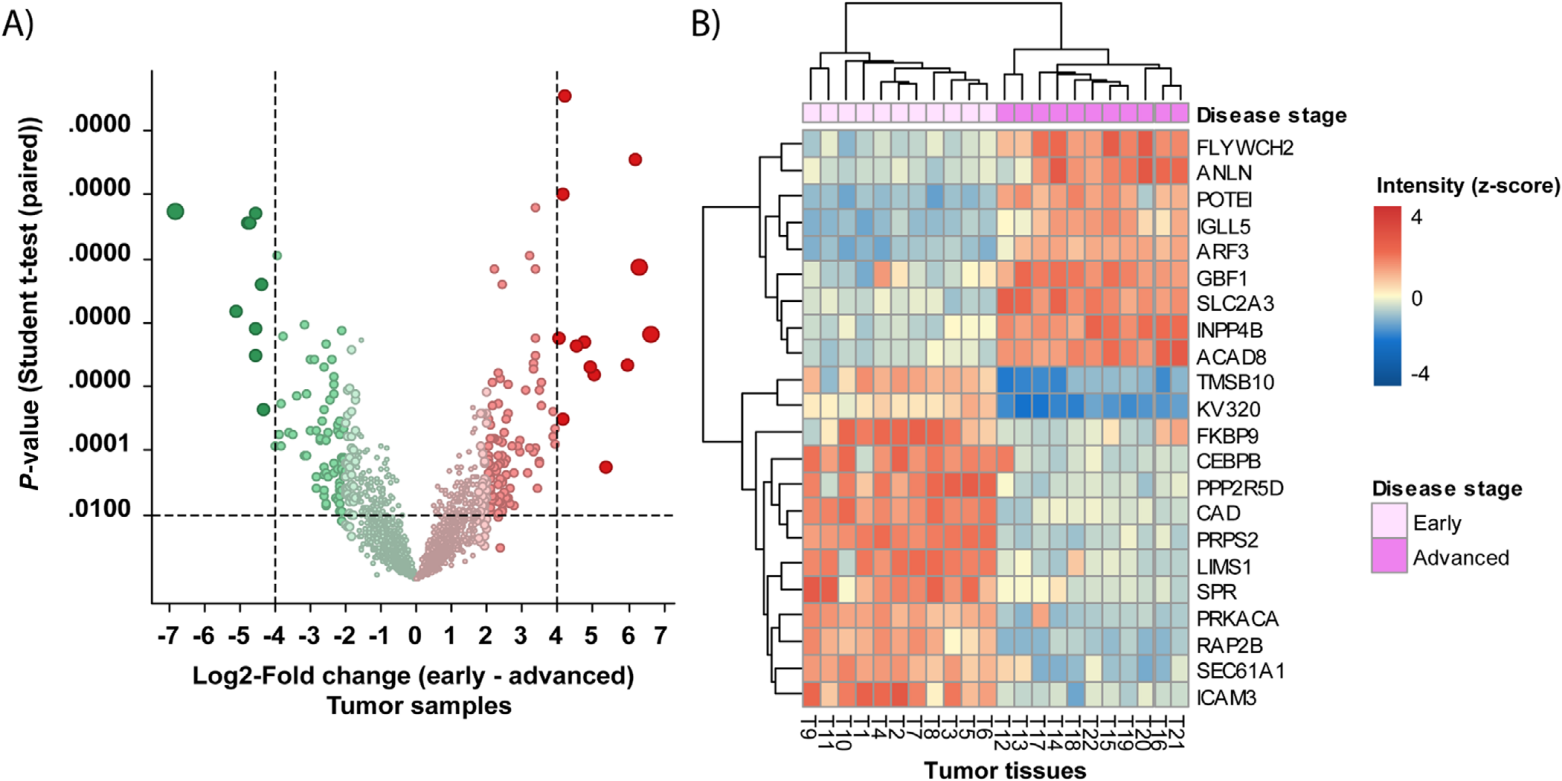
Comparison of early‐ and advanced‐stages in tumor tissue. **A,** Volcano plot of log_2_‐fold change (FC) versus *P*‐value *t*‐test. Points alter in size according to the magnitude of the fold change. **B,** Heat map generated by hierarchical clustering of the most altered proteins. The heat map shows a clear separation of adjacent normal and tumor tissues (–4 ≥ log_2_ FC ≥ 4, adj. *P* < .01). Blue and red correspond to decreased and increased expression levels, respectively

To characterize early‐ and advanced‐stage ADC, the proteins that were differentially expressed between the early and advanced tumor samples were determined. In total, 84 and 71 proteins were down‐ and upregulated, respectively, in advanced tumor tissue compared to early tumor tissue (–2 ≥ log_2_ FC ≥ 2; *q*‐value < .01) (see Table S2D). The proteins with the highest altered expression (–4 ≥ log_2_ FC ≥ 4) are given in Table S2C and displayed as a heat map in Figure 5B.

Reactome pathway terms that are overrepresented in the 71 upregulated proteins were EPH‐ephrin signaling (AP2A1, MYL9, and ACTB), signaling by Rho GTPases (MYL9, ACTB, GMIP, NCF4, ARHGEF1, and PIN1), and beta‐catenin independent Wnt‐signaling (AP2A1, PLCB2, PSMD13, and PSMB5). All of which have been implicated in tumorigenesis (Table S9).

Moreover, pathways that are associated with the upregulated proteins are involved in tumorigenesis. These include the VEGFA‐VEGFR2 pathway, insulin receptor signaling cascade, beta‐catenin independent WNT signaling, signaling by Rho GTPases, and the RAF/MAP kinase cascade.

Reactome pathways associated with the downregulated proteins are involved in the extracellular matrix organization (ITGAX, FBN1, MMP2, ICAM3, COL6A2, and FGA), integrin cell surface interactions (ITGAX, FBN1, ICAM3, COL6A2, and FGA), degradation of the extracellular matrix (FBN1, MMP2, and COL6A2), and ECM proteoglycans (ITGAX and COL6A2) (Table S10).

### 3.5 Early‐stage ADC‐specific proteins

At the protein level, distinct changes occur during tumor progression. Such changes range from altered expression and differential protein modification to changes in protein activity and altered localization. Detecting stage‐specific changes in cancer proteomes may assist in identifying potential biomarkers that enable detection of the disease at an earlier stage.^27^ Therefore, from the list of 67 proteins (Table S5) that were exclusively quantified in all early‐stage ADC samples (but not in the matched normal samples), 18 proteins were further assessed based on the differential expression between early‐ and advanced‐stage ADC (Table 2). Out of 18, seven proteins were quantified in early but not in advanced ADC samples (ARAP1, ZFR, EDC3, HMOX2, NT5C3A, PRPS2,and ICAM3; referred as ON/OFF in Table 2) and 11 proteins were overexpressed in early‐stage ADC (MCM6, SNRNP70, CDC5L, RBM12, RBM17, S100A14, THUMPD1, MX1, UBE2H, PXDN, and SPR; adj. *P*‐value < .01).

**TABLE 2:** Top 18 potential early stage‐specific proteins. These proteins were quantified in all tumor samples from early‐stage ADC but not in their matched normal samples. The log_2_‐fold changes (FC) indicate that their intensities were higher in early ADC than in advanced ADC. ON/OFF: proteins quantified in early but not in advanced stage ADC samples. All proteins presented an adj. *P*‐value < .01 (T‐test_Early vs Advanced ADC)

Gene symbol	Protein name (UniProt accession)	Log_2_ FC (Early/Advanced ADC)	Known functions	Relation to cancer
ARAP1	ArfGAP With RhoGAP Domain, Ankyrin Repeat, and PH Domain 1 (Q96P48)	ON/OFF	Coordinates the membrane and actin remodeling involved in cell movement.	Prevents EGFR degradation.^88^, ^89^
ZFR	Zinc finger RNA‐binding protein (Q96KR1)	ON/OFF	Regulates alternative pre‐mRNA splicing and plays an essential role in cell growth.	Potential therapeutic target in human pancreatic cancer.^90^ Involved in NSCLC tumor growth and metastasis.^91^
EDC3	Enhancer Of MRNA decapping 3 (Q96F86)	ON/OFF	Component of mRNA decapping complex and involved in mRNA decay.^93^	–
HMOX2	Hem oxygenase 2 (P30519)	ON/OFF	Heme degradation to biliverdin, iron, and carbon monoxide, cytoprotective and anti‐inflammatory function.^107^	May be associated with the prognosis of bladder cancer.^92^
NT5C3A	5′‐Nucleotidase, cytosolic IIIA (Q9H0P0)	ON/OFF	Catalyzes the dephosphorylation of pyrimidine 5′ monophosphates.	–
PRPS2	Phosphoribosyl pyrophosphate synthetase 2 (P11908)	ON/OFF	Plays a central role in the synthesis of pyrimidines and purines.	Promoted increased nucleotide biosynthesis in Myc‐transformed cells.^48^
ICAM3	Intracellular adhesion molecule 3 (P32942)	ON/OFF	Constitutively and abundantly expressed by all leucocytes and may be the most important ligand for LFA‐1 in the initiation of the immune response.	Promoted cancer cell migration and invasion.^106^ Induced cancer cell proliferation in vitro in lung cancer.^44^ Stimulated cancer cell migration/invasion via ICAM‐3/Akt/CREB/MMP pathway in NSCLC cells.^45^
MCM6	Minichromosome maintenance complex component 6 (Q14566)	1.29	Essential component for DNA replication.	Overexpressed in NSCLC.^108^ Component of a 6‐protein panel, which is a potential biomarker for the early detection of lung cancer in bronchial brushings.^109^
SNRNP70	Small nuclear ribonucleoprotein U1 subunit 70 (P08621)	1.60	Component of the spliceosome.	Its gene was found upregulated in lung ADC.^110^
CDC5L	Cell division cycle 5 like (Q99459)	1.66	DNA‐binding protein involved in cell cycle control, pre‐mRNA splicing, and DNA damage response.	Implicated in colorectal cancer,^111^ prostate cancer,^112^ and hepatocellular carcinoma.^113^
RBM12	RNA binding motif protein 12 (Q9NTZ6)	1.90	RNA‐binding protein.	Upregulated in meibomian cell carcinoma.^114^
RBM17	RNA binding motif protein 17 (Q96I25)	1.99	Component of the spliceosome complex.	Overexpressed in numerous carcinomas, including lung.^28^
S100A14	S100 calcium‐binding protein A14 (Q9HCY8)	2.01	Calcium‐binding protein involved in cell proliferation and differentiation.	Implicated in tumorigenesis in various cancer types.^115^, ^116^, ^117^, ^118^ Overexpressed in lung ADC1;^119^ its expression correlated with invasion of lung ADC cells.^120^
THUMPD1	THUMP domain containing 1 (Q9NXG2)	2.30	tRNA‐binding adapter to mediate NAT10‐dependent tRNA acetylation.^121^	Overexpressed in breast cancer and promoted metastasis.^122^
MX1	MX dynamin like GTPase 1 (P20591)	2.31	Play a pivotal role in the type I interferon‐mediated response against viral infections.	Potential bone metastasis biomarker in lung cancer.^123^
UBE2H	Ubiquitin conjugating enzyme E2 H (P62256)	3.35	E2 ubiquitin‐conjugating enzyme.	Plays a role in HCC development.^124^, ^125^ MET‐UBE2H fusion a novel resistance gene arrangement to EGFR‐TKI in lung ADC.^126^
PXDN	Peroxidasin (Q92626)	3.54	Heme‐containing peroxidase involved in the formation and stabilization of extracellular matrix.	Has been detected in several cancer types.^43^ Its overexpression is associated with poor prognosis in ovarian cancer.^127^
SPR	Sepiapterin reductase (P35270)	5.03	Catalyzes the last step of BH4 biosynthesis.	Over expression of SPR mRNA correlated with poor prognosis in neuroblastoma patients.^41^

**FIGURE 6:**
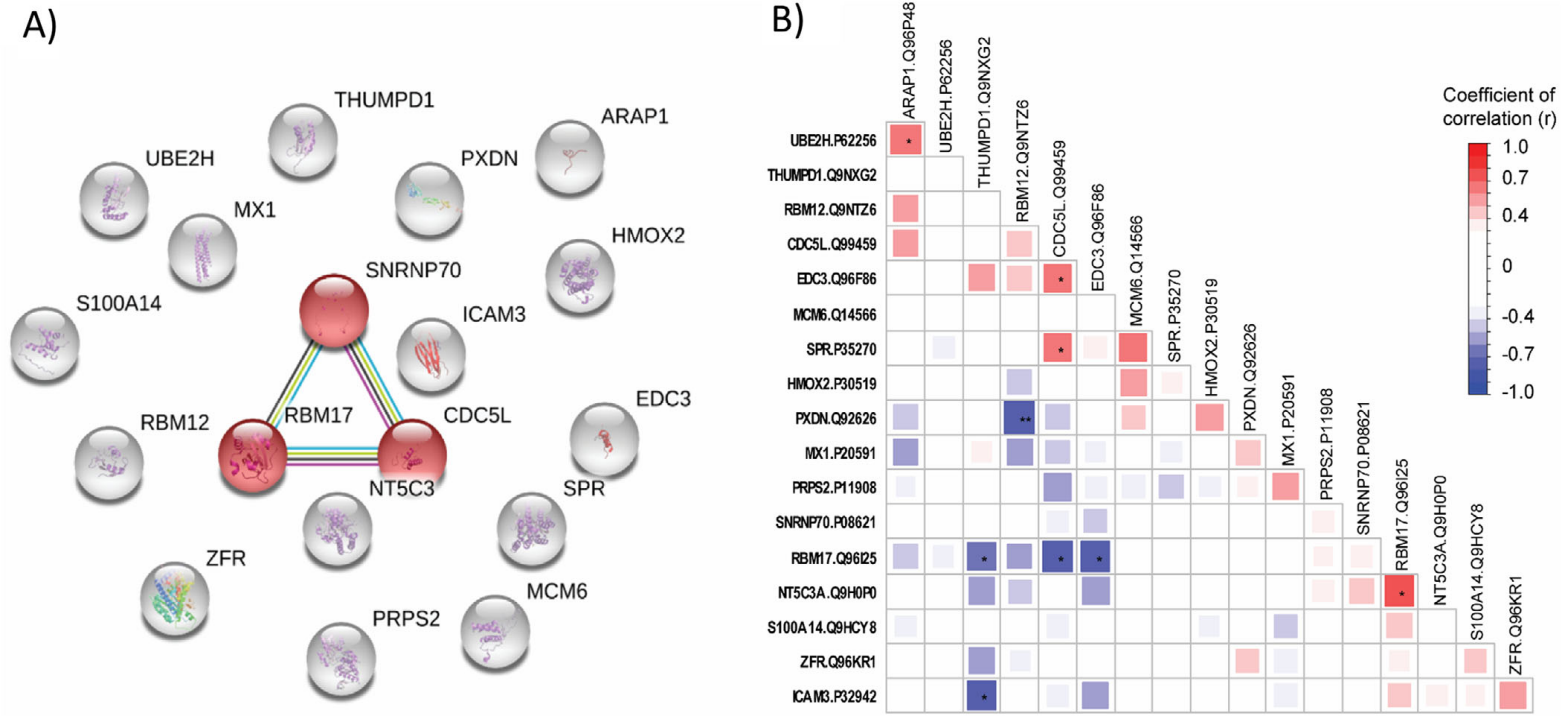
Protein‐protein interaction and correlation for the 18 early‐stage ADC‐specific proteins. **A,** Protein network from STRING. Shown in red are the significant interactions (FDR < .05 with the highest confidence threshold). **B,** Spearman correlation. The color scale represents the strength of the correlation (*r*) (white to red, positive correlation; white to blue, negative correlation). *P < .05; **P < .01

To analyze the associations among these 18 proteins (quantified in all early‐stage samples), the STRING database was used to retrieve interacting genes/proteins. Following the STRING network analysis, only three proteins (all components of the spliceosome) showed a strong association with one another (Figure 6A). SNRNP70 (small nuclear ribonucleoprotein U1 subunit 70) associates with U1 snRNA and is essential for the 5′ splice site selection. RBM17 (RNA‐binding motif protein 17) is involved in the regulation of alternative splicing and is frequently overexpressed in various solid tumors.^28^ CDC5L (cell division cycle 5 like) is a core component of the spliceosomal complex and essential for pre‐mRNA splicing^29^ and involved in DNA damage repair.^30^ To date, several alterations in mRNA metabolism have been reported in lung cancer suggesting that mRNA metabolism‐related proteins are involved in the pathology of the disease.^31^, ^32^

To further examine the relationship between the proteins, a Spearman correlation analysis was performed using the 11 values from early tumor stage. Only nine significant correlations were observed among the 18 proteins. Positive correlations were apparent between RBM17 and NT5C3A; ARAP1 and UBE2H; CDC5L and EDC3; and CDC5L and SPR. Negative correlations were observed between RBM12 and PXDN; RBM17 and CDC5L; RBM17 and EDC3; THUMPD1 and ICAM3; and RBM17 and THUMPD1 (Figure 6B; Table S11). Interestingly, there were four significant correlations for the protein RBM17. Nevertheless, no evidence from the literature was found supporting an association between RBM17 and NT5C3A, and EDC3 and THUMPD1. Taken together, the expression levels of the 18 proteins did show the same dynamics between the tumor stages, namely, upregulated in early‐stage ADC and downregulated in advanced ADC tissues. These proteins, however, did not show any clear association with one another, neither with published data nor with our values. To enrich the number of related proteins, further research with a larger patient cohort is a necessity.

## 4 DISCUSSION

In this study, the proteomes of early‐ and advanced‐stage lung ADC plus normal adjacent tissue were generated. The identified proteins were described and the most DEPs between normal and tumor samples and between early‐ and advanced‐stage tumor tissues were discussed. The generated data were also compared to currently available literature in the context of improving our understanding of the molecular basis of lung ADC.

Over the past decade, considerable efforts have been made to discover potential protein biomarkers that can be used to detect and monitor the progression of lung cancer. Despite these efforts, however, lung cancer remains the leading cause of cancer‐related mortality worldwide.^33^ Thus, it is critical to obtain more knowledge on the molecular complexity of lung ADC.

MS‐based proteomics enables the analysis of dynamic and complex systems in biology. Label‐free proteomic analyses not only provide a list of identified proteins but also enable the quantitation of relative changes in protein expression levels between sets of samples. Both prognostic and predictive biomarkers for lung cancer found in tissue, cells, blood, or other body fluids have been discovered using MS‐based proteomics approaches (reviewed by Cheung and Juan^9^). The application of proteomic analyses of paired tumor and control tissue can aid in the investigation of pathological processes in lung ADC. In this study, a label‐free proteomics workflow was applied. The protocol required minimal sample manipulation and was versatile and cost‐effective.

During the analysis of the proteomic profiles of the paired tumor and normal lung tissues, an observation was made whereby the number of identified proteins was higher in the cancer tissues compared to the matched controls (average protein number in normal tissue: 3272; average protein number in ADC: 4336).

### 4.1 DEPs in early‐ and advanced‐stage ADC

Many of the DEPs between normal and tumor samples (early stage) have been previously implicated in tumorigenesis in the literature. The RNA‐binding protein QKI is a key regulator of alternative splicing in lung cancer and has been frequently reported as downregulated in lung cancer. Downregulation is associated with poor prognosis.^34^ SAFB (scaffold attachment factor B) belongs to the nuclear matrix family of proteins and low‐protein expression is significantly associated with worse overall survival in breast cancer patients who did not receive adjuvant therapy.^35^ A decreased level of the protein ANK1 (ankyrin 1) that is known to regulate cell shape and membrane integrity has been observed in lung ADC.^36^ Ion channels are involved in diverse biological functions and it is known that dysregulated expression of such proteins contributes to tumor progression.^37^ Recently, the gene expression profile of 37 ion channels was analyzed in lung ADC. Several ion channels including CLIC3 (chloride intracellular channel 3) were downregulated.^38^

BH4 (tetrahydrobiopterin) synthesis promotes both endothelial cell proliferation, migration, and tube formation in vitro and angiogenesis in tumor xenografts.^39^ Moreover, BH4 induces A549 cell proliferation and migration via the activation of Akt and p70^S6K^ signaling.^40^ In tumorigenesis, relatively little is known about the role of SPR (sepiapterin reductase) that catalyzes the last step of BH4 biosynthesis. Recently, Lange et al^41^ reported an oncogenic role for SPR in neuroblastoma and found that overexpression of SPR mRNA correlates with poor prognosis in patients. DNM1L (dynamin 1‐like protein) is essential for normal mitochondrial function and is upregulated in several cancer types including lung cancer.^42^ PXDN (peroxidasin) is involved in the formation and stabilization of the ECM and has been detected in several types of cancer.^43^ ICAM3 (intracellular adhesion molecule 3) has been shown to induce cancer cell proliferation in vitro in lung cancer^44^ and promote cancer cell migration and invasion.^45^ SRSF3 (serine arginine‐rich splicing factor 3) is a well‐known RNA processing protein that is overexpressed in several cancer types and involved in tumor maintenance.^46^ CPS1 (carbamoyl‐phosphate synthase 1) is a multi‐domain mitochondrial enzyme that is involved in arginine and pyrimidine metabolism and was shown to be statistically significantly associated with poor overall survival in stage I lung ADC.^47^ Cancer cells have the ability to alter cellular processes to sustain an enhanced metabolism for increased cell growth and proliferation. Recently, it has been reported that PRPS2 (phosphoribosyl pyrophosphate synthetase 2), a protein that plays a central role in the synthesis of pyrimidines and purines, promoted increased nucleotide biosynthesis in Myc‐transformed cells.^48^ PAIP1 (poly(A)‐binding protein‐interacting protein 1) was detected only in tumor tissue and overexpression of PAIP1 in vitro stimulates translation.^49^

Among the dysregulated protein between normal and tumor samples (advanced stage), INPPB4 (type II inositol 3,4‐bisphosphate 4‐phosphatase) has been previously identified as a tumor suppressor and expression of this protein is reduced in several types of cancers including breast, ovarian, and prostate.^50^, ^51^, ^52^ A recent report, however, indicated that INPPB4 promotes an oncogenic signaling pathway in breast cancer.^53^

QSOX1 (quiescin sulfhydryl oxidase 1) has an emerging role in cancer and was shown to be overexpressed in several malignancies including breast, pancreas, and prostate cancer.^54^, ^55^ A general consensus is emerging that QSOX1 overexpression is important during tumor cell invasion, facilitating tumor cell migration at the tumor‐stroma interface.^56^ Recently, it was reported that LMAN2 (vesicular integral‐membrane protein VIP36) is overexpressed in gastric cancer.^57^

### 4.2 Early versus advance ADC

Some of the upregulated proteins in early‐stage ADC were ribosomal proteins, mRNA‐splicing factors, and translation factors. Emerging evidence suggests that ribosomal proteins not only play essential roles in protein synthesis, but are also involved in cancer tumorigenesis.^58^, ^59^, ^60^ Recently, it was demonstrated that RPS15A (ribosomal protein S15A) expression is increased in lung ADC tissue and knockdown of RPS15A inhibited cancer cell growth and induced apoptosis.^61^ It has been suggested that RPS6 (ribosomal protein 6) is overexpressed in NSCLC and downregulation thereof inhibits cell growth.^62^ It is well known that aberrant mRNA splicing contributes to cancer progression and the expression of splicing factors are altered in tumor tissues.^63^, ^64^, ^65^, ^66^ Gout et al^67^ suggested that global deregulation of pre‐mRNA splicing factors occurs during lung tumorigenesis. These researchers demonstrated that SRSF1 and SRSF2 (serine and arginine‐rich splicing factor 1 and 2) and SRPK1 and SRPK2 SRSF protein‐specific kinases are upregulated in NSCLC. In our study, SRSF7 and SRSF2 were statistically significantly up‐ and downregulated in advanced ADC, respectively (*q*‐value < .01), but did not meet the established criteria for FC (log_2_ FC = 1.126; log_2 _FC = –1.618, respectively). On the other hand, a prognostic mRNA splicing signature was identified by Gout et al^67^ in lung ADC and splicing networks were revealed. Together, these could reveal novel cancer drivers and provide new insight in lung ADC etiology.^68^ Translational regulation is a critical process for maintaining cellular homeostasis and allowing rapid cell adaptation under stress. Therefore, it is not suprising that dysregulated translation plays an important role in tumorigenesis.^69^ Expressions of specific subunits of EIF3 are altered in a variety of human tumors. Elevated expression of EIF3A was observed in lung cancer^70^ and overexpression of the eucaryotic translation inition factor 4E and 4H in lung cancer has also been reported.^71^, ^72^ Moreover, phase II clinical trials of an EIF4E antisense oligonucleotide (ASO) combined with carboplatin and paclitaxel for NSCLC are ongoing (NCT01234038).

Several of the upregulated proteins in advanced ADC samples have been previously reported in the literature as involved in tumor progression. Serum levels of POTE (POTE ankyrin domain family member I), a paralog of POTEI (POTE ankyrin domain family member I), in NSCLC patients are associated with TNM stage (tumor extension, nodal status, and metastatic spread incorporated into the staging system).^73^ ARF3 (ADP‐ribosylation factor 3) belongs to the human ADP‐ribosylation factor gene family and is involved in vesicular trafficking. The actin‐binding protein ANLN (actin‐binding protein anillin) is a ubiquitously expressed protein required for cytokinesis. Upregulation of ANLN expression is frequently observed during cancer development, growth, and progression.^74^, ^75^ Moreover, it has been reported that nuclear ANLN protein expression in lung cancer tissue is significantly correlated with poor survival.^76^ Aberrant expression of SLC2A3 (solute carrier family 2, facilitated glucose transporter member 3) has been reported in gastric, testicular, ovarian, and NSCLC.^77^, ^78^ Furthermore, it has been shown that SLC2A3 induces tumor cell proliferation in NSCLC.^79^

On the other hand, extracellular matrix organization (ECM) was associated with proteins downregulated in advanced ADC. It is well‐known that cancer development and progression is associated with ECM. The ECM is a highly dynamic structure and a major component of the microenvironment. Abnormal ECM dynamic leads to dysregulated cell proliferation and migration.^80^, ^81^ Collagen is the most abundant constituent of the ECM and increased or decreased expression of collagen can contribute to increased malignancy.^82^, ^83^, ^84^ CEACAM6 overexpression was previously reported in various types of cancer including NSCLC.^85^ Dysregulated overexpression of CEACAM6 is oncogenic and associated with an invasive tumor phenotype.^86^

The most abundant protein observed in early‐stage tumor tissues was FKBP9 (peptidyl‐prolyl cis‐trans isomerase FKBP9) (upregulated in early‐stage tumor tissues). This protein belongs to the family of peptidyl‐prolyl isomerases (PPIase) that catalyze peptidyl‐prolyl cis‐trans isomerization and function as molecular chaperones that play a crucial role in tumorigenesis. PPIases mediate conformational modifications in proteins that modulate signaling pathways and are overexpressed in a variety of tumors.^87^ Development of isoenzyme‐specific inhibitors has been in the focus of recent biomedical research.

### 4.3 Early‐stage ADC‐specific proteins

The detection of proteomic changes that occur during tumor progression may aid in identifying potential stage‐specific markers for diagnosis. The prognosis for lung cancer patients is strongly related to the stage of the disease at the time of diagnosis. Seven of the 18 early stage‐specific proteins were identified only in the early stage ADC samples (referred as ON/OFF in Table 2). ARAP1 (ArfGAP with RhoGAP domain, ankyrin repeat, and PH domain 1) prevents EGFR degradation^88^, ^89^ and thus may increase the oncogenic capabilities of the cells. ZFR (zinc finger RNA‐binding protein) is involved in the regulation of alternative pre‐mRNA splicing and plays an essential role in cell growth and maybe a potent therapeutic target in human pancreatic cancer.^90^ In a recent study, it was demonstrated that ZFR is involved in NSCLC tumor growth and metastasis.^91^ Among the 18 selected proteins, 11 were also observed in the advanced‐stage tumor samples (Table 2). These were downregulated in advanced‐stage ADC suggesting that expression thereof may be early stage specific.

Several of the proteins quantified exclusively the early‐stage tumor tissues have been implicated in cancer. HMOX2 (heme oxygenase 2) may be associated with the prognosis of bladder cancer.^92^ ZFR has been reported to play an important role in DNA binding and plays an essential role in cell growth and maybe a potent therapeutic target in human pancreatic cancer.^90^

Although several of the selected early‐stage ADC‐specific proteins have been reported to play a role in tumorigenesis in various cancer types (Table 2), EDC3 and NT5C3A have not been implicated in lung cancer. EDC3 (enhancer of mRNA decapping 3) is a component of the mRNA decapping complex and important for mRNA stability and decay.^93^ Removal of the 5′ end cap structure from mRNAs is a crucial control step in the cytoplasmic degradation of mRNAs, and thus an essential process in posttranscriptional regulation of gene expression.^94^, ^95^ Alterations in the protein expression level of specific mRNA decapping factors may lead to a deregulated mRNA decay pathway and potentially contribute to tumorigenesis.^96^, ^97^ NT5C3A (5′‐nucleotidase, cytosolic IIIA) is a member of the 5′‐nucleotidase family and participates in nucleotide homeostasis by catalyzing the dephosphorylation of pyrimidine monophosphates.^98^ It has been demonstrated that NT5C3A plays a critical role in the metabolism of, and resistance to, chemotherapeutic nucleoside analogues such as gemcitabine and cytosine arabinoside.^99^ Recently, it has been shown that NT5C3A acts as a negative regulator of the inflammatory cytokine response.^100^ Aberrations in the cytokine response pathways can alter gene expression subsequently leading to tumor progression.^101^

These potential stage‐specific proteins require further additional investigation across a larger patient cohort.

### 4.4 Proteomics studies with lung ADC samples

The survival rate of lung cancer patients strongly correlates with tumor stage. Therefore, improving diagnostic strategies for early tumor detection may lead to an increase in patient survival. An approach using iTRAQ labeling recently identified 133 protein candidates from paired lung ADC with differing degrees of lymph node involvement.^102^ Six potential biomarkers that were overexpressed in ADC tissue comparing to adjacent normal tissues were further validated (ERO1L, NARS, PABPC4, RCC1, RPS25, and TARS). In addition, ERO1L and NARS were positively associated with lymph node metastasis.^102^ Employing a gel‐free proteomic approach, Kawamura et al identified 81 proteins that were associated with stage IA and IIIA lung ADC from FFPE tissues. Napsin‐A (NAPSA) and anterior gradient protein 2 homolog (AGR2) were identified as potential stage‐specific candidates for stage IA and stage IIIA lung ADC.^103^ In a recent study, zyxin (ZYX)—a novel potential early diagnostic biomarker—was identified from plasma by LC‐SRM.^104^

## 5 CONCLUSIONS

We demonstrated that the proteomic workflow used here enabled a clear distinction between lung ADC and matched normal tissue samples and also between early‐ and advanced‐stage tumor specimens. Our large‐scale, label‐free proteomic dataset of histologically well‐classified lung ADC may provide a deeper insight into the molecular mechanisms underlying lung ADC progression. As expected, the complexity of the proteome from the tumor was higher than the normal tissue proteome. Thirty‐three and 39 DEPs were identified in early‐ and advanced‐stage ADC, respectively (adj. *P*‐value < .01, FC ≥ 4). Although several of these proteins have been indicated in tumorigenesis and progression, none had been previously reported for lung ADC. Based on the biological functions of these proteins, the results revealed that the most enriched pathways are involved in mRNA metabolism. Furthermore, 18 potential early stage‐specific proteins were identified that may be useful as predictive markers for lung ADC. To validate the findings of this study, a larger sample size/patient cohort and/or orthogonal methods are imperative.

## CONFLICT OF INTEREST

The authors declare no conflict of interest.

## Supporting information

Supporting Information

Supporting Information

Supporting Information

Supporting Information

Supporting Information

## Data Availability

The mass spectrometry proteomics data have been deposited to the ProteomeXchange Consortium via the PRIDE^105^ partner repository with the dataset identifier PXD019259.
