# Supplementary figures and images for: Proteomic analysis enables distinction of early‐ versus advanced‐stage lung adenocarcinomas
Source: Clin Transl Med. 2020 Jun 14;10(2):e106. doi: 10.1002/ctm2.106 (PMC7403673; doi:10.1002/ctm2.106)

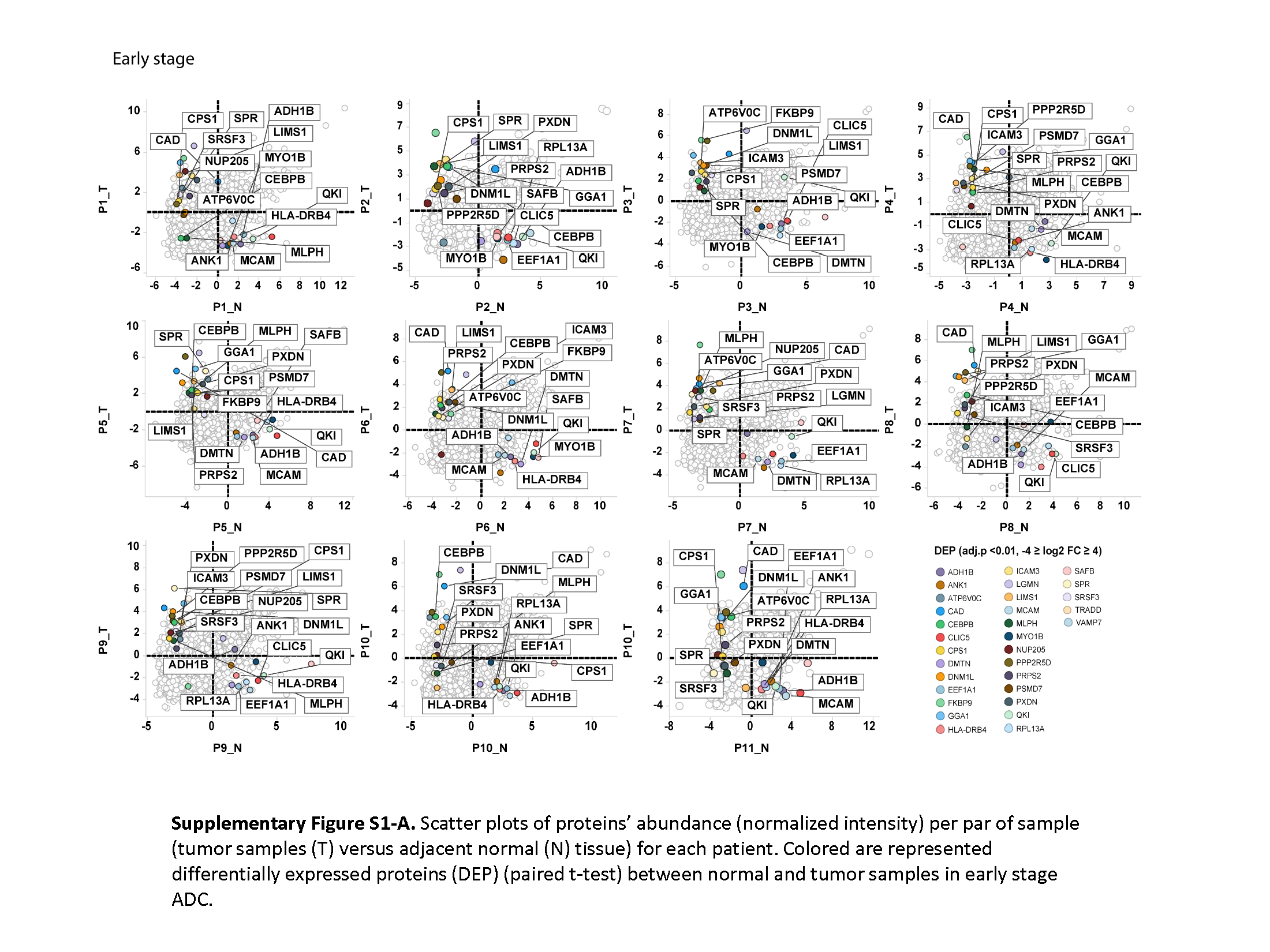

Supplement: Supplementary file 1 — Supporting Information [file CTM2-10-e106-s001.tiff]

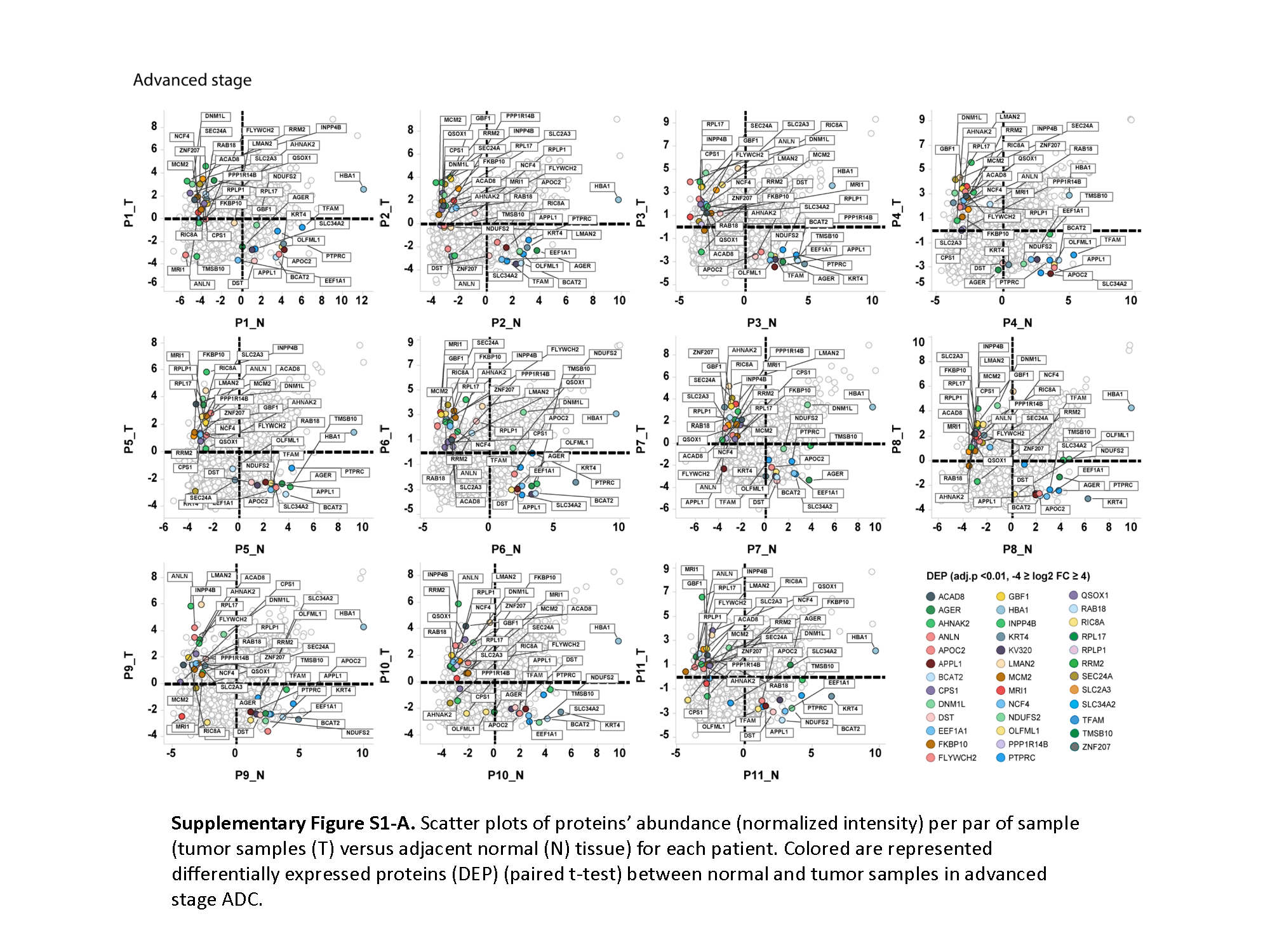

Supplement: Supplementary file 2 — Supporting Information [file CTM2-10-e106-s002.tiff]
