# Supplementary material for: Proteomic analysis enables distinction of early‐ versus advanced‐stage lung adenocarcinomas
Source: Clin Transl Med. 2020 Jun 14;10(2):e106. doi: 10.1002/ctm2.106 (PMC7403673; doi:10.1002/ctm2.106)
Supplement: Supplementary file 3 — Supporting Information [file CTM2-10-e106-s003.pdf]

Supplementary File F1

Hematoxylin and Eosin (H&E) stained sections of tumor and adjacent normal lung tissue samples. Tumor (right) and normal adjacent (left). Numbers correspond to sample ID number. Scale bar = 100 µm.

Patient characteristics

patient ID	sample ID normal	sample ID tumor	age at the diagnosis	gender	smoker 0= never, 1=current, 2=former	histology	tumor %	pT(TNM)	pN(TNM)	pM(TNM)	stage	grade
P1	1	2	65	F	2	ADC	n/a	2	1	0	IIa	n/a
P2	3	4	70	M	2	ADC	95	2b	0	0	IIa	n/a
P3	7	8	55	M	1	ADC	75	3	0	0	IIb	n/a
P4	9	10	56	M	1	ADC	35	1b	0	0	Ia	III
P5	11	12	70	M	2	ADC	15	2b	0	0	IIa	n/a
P6	15	16	50	F	1	ADC	55	1a	0	0	Ia	III
P7	17	18	55	F	2	ADC	40	1b	0	0	Ia	II-III
P8	45	46	63	M	1	ADC	80	1a	0	0	Ia	II
P9	43	44	60	F	1	ADC	90	2b	0	x	IIa	II
P10	47	48	46	M	0	ADC	60	2b	1	x	IIb	III
P11	49	50	64	M	2	ADC	65	3	0	x	IIb	n/a
P12	19	20	57	M	2	ADC	70	2a	2	1	IV	II
P13	21	22	47	M	2	ADC	70	2a	2	x	IIIa	II
P14	23	24	60	F	2	ADC	70	1b	2	x	IIIa	II
P15	25	26	57	M	2	ADC	45	2	2	x	IIIa	II
P16	27	28	60	F	2	ADC	50	2a	2	x	IIIa	II
P17	29	30	74	M	2	ADC	85	2a	2	x	IIIa	II
P18	31	32	64	F	1	ADC	30	2b	2	x	IIIa	III
P19	35	36	61	M	1	ADC	95	2b	2	x	IIIa	III
P20	39	40	64	M	n/a	ADC	85	3	1	x	IIIa	III
P21	37	38	58	F	1	ADC	90	2a	0	1	IV	III
P22	41	42	68	F	1	ADC	80	3	1	0	IIIa	n/a

3

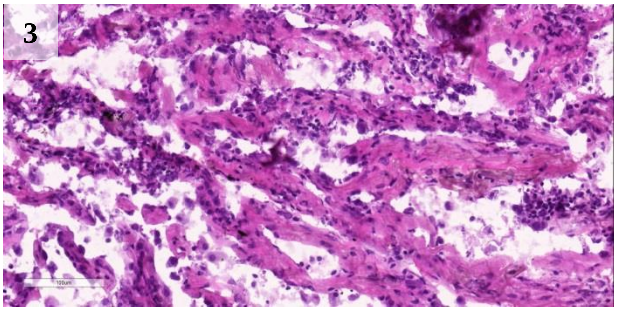

4

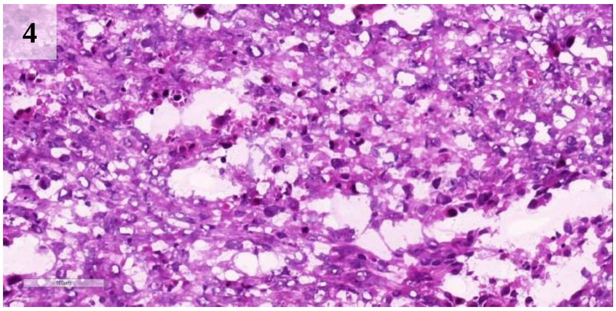

7

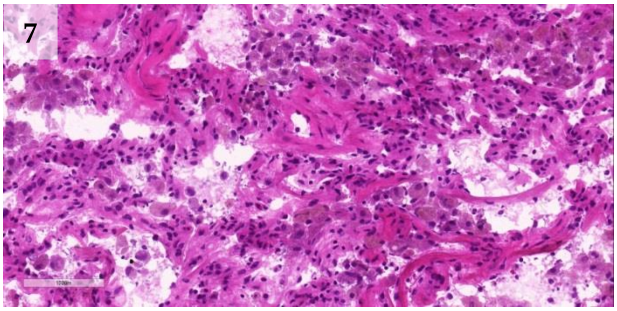

8

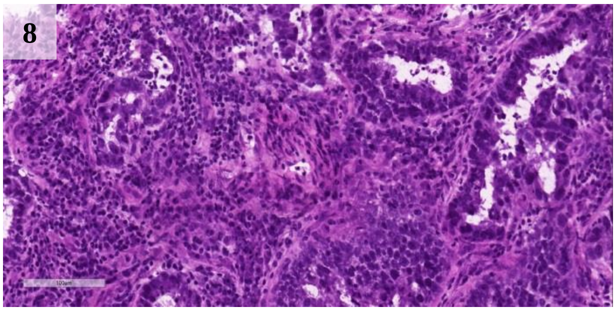

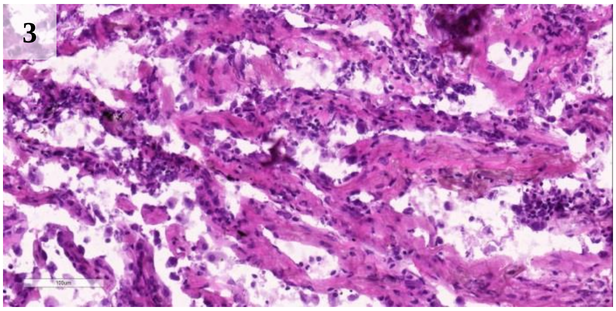
3
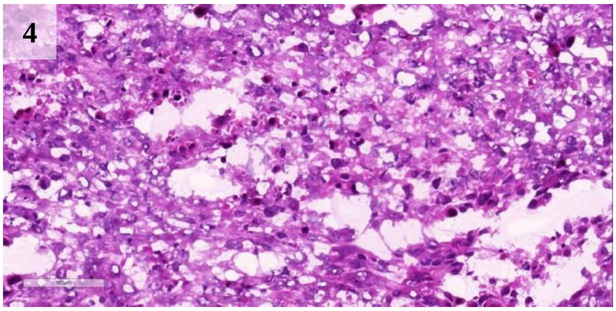
4
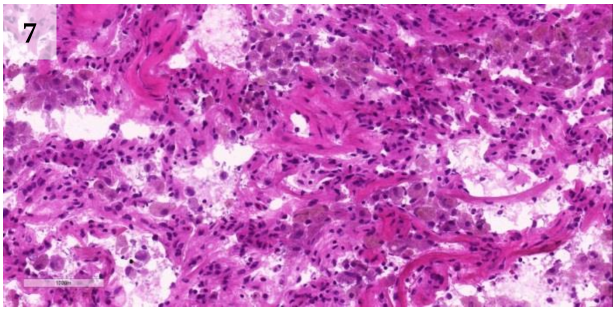
7
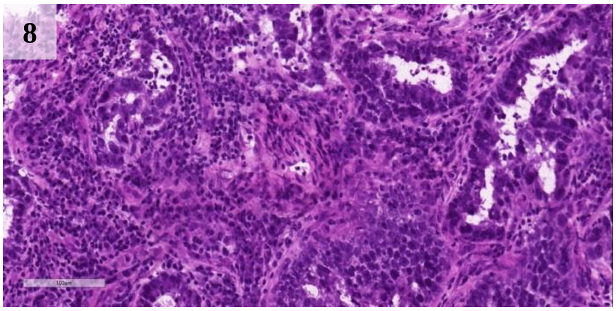
8
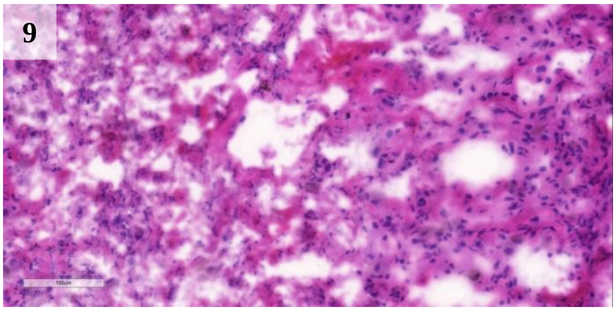
9
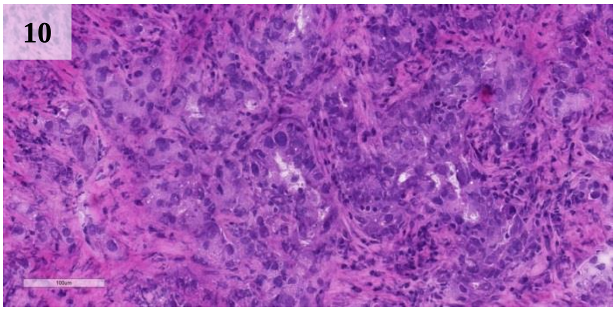
10
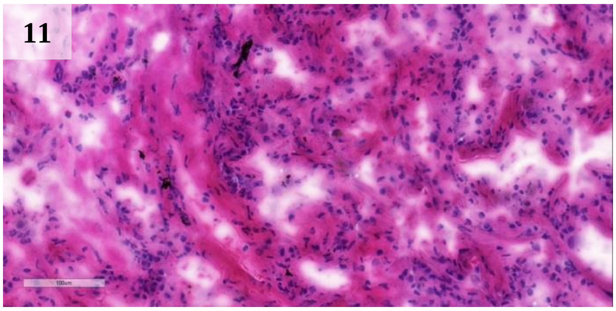
11
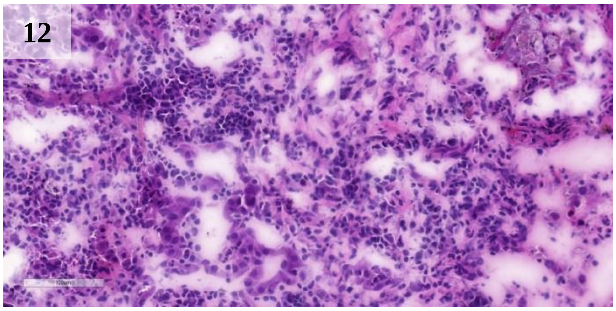
12
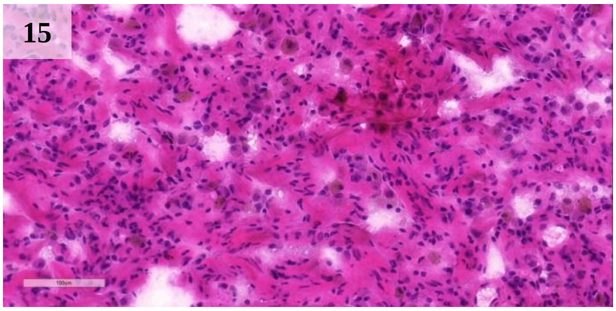
15
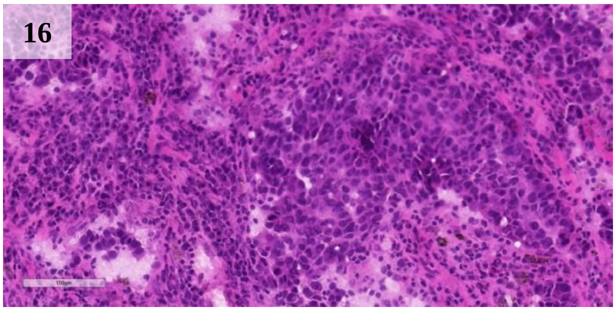
16

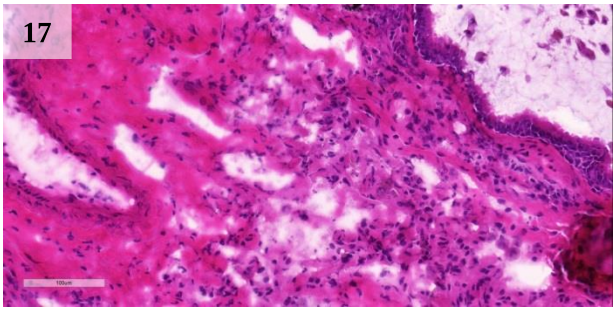
17

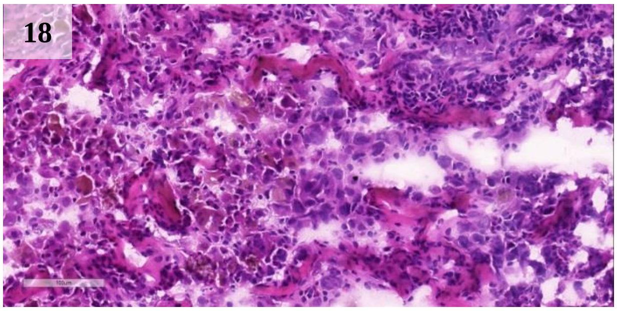
18

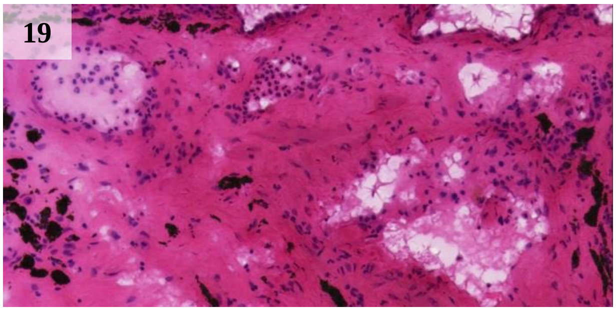
19

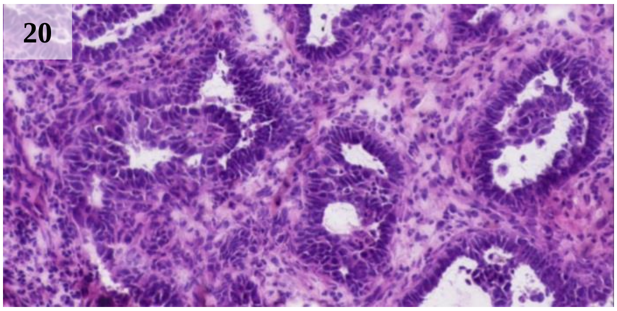
20

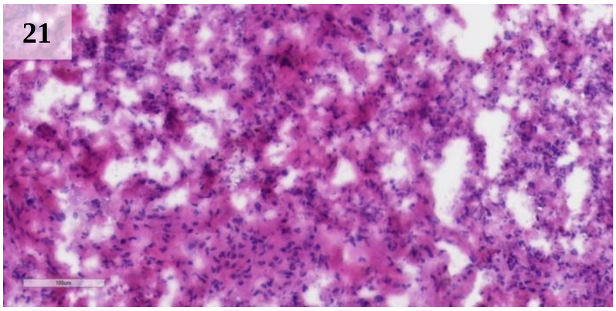
21

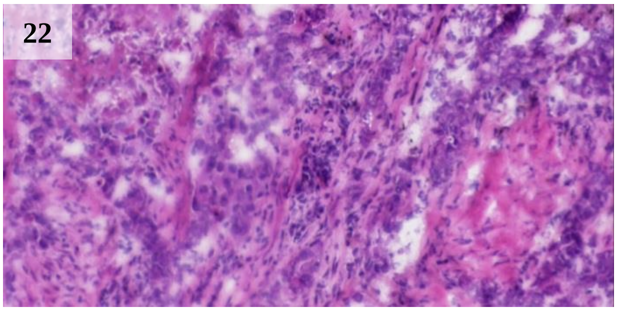
22

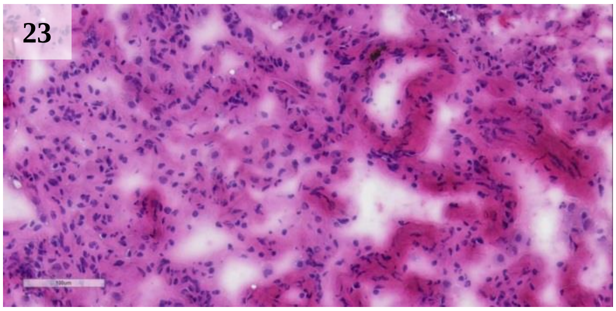
23

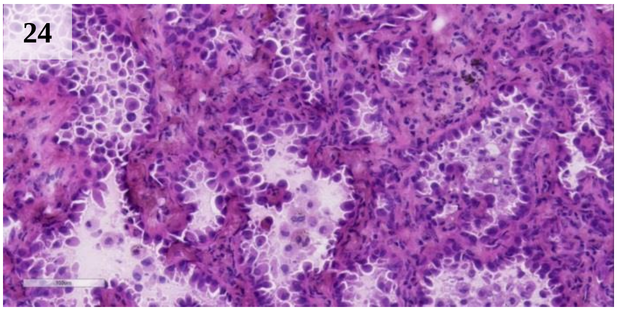
24

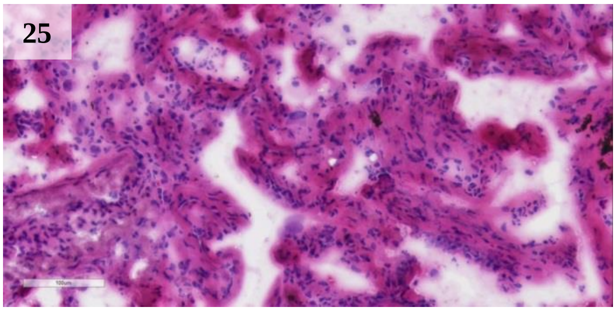
25

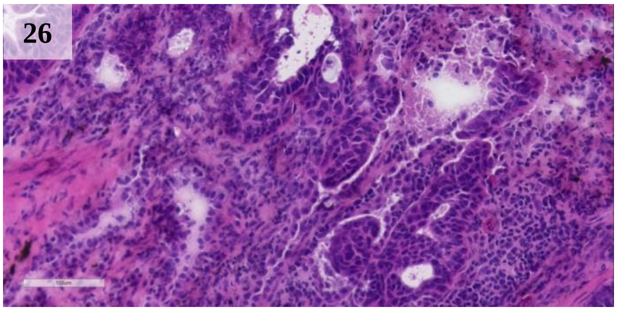
26

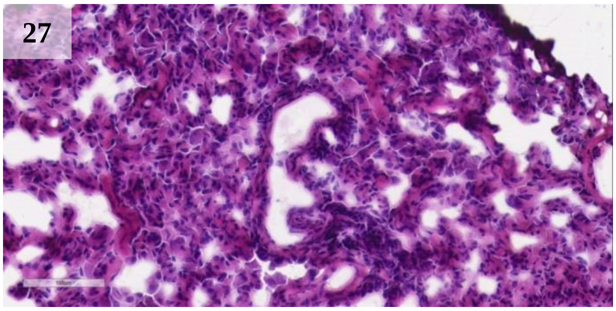
27

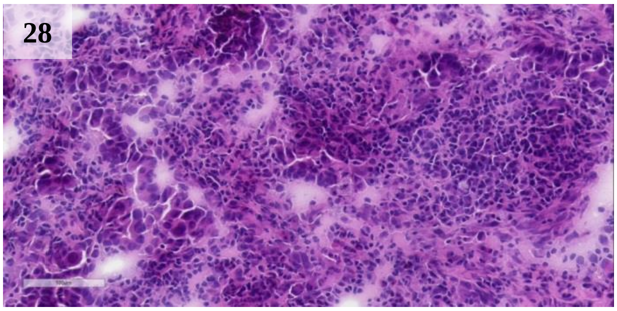
28

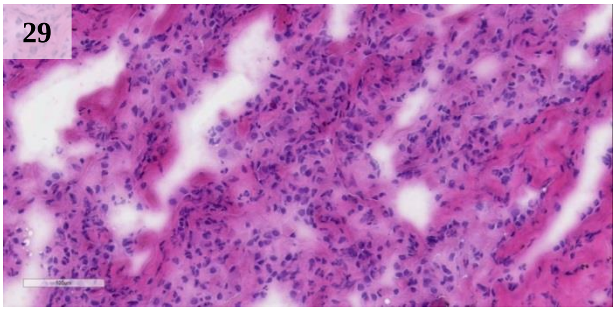
29

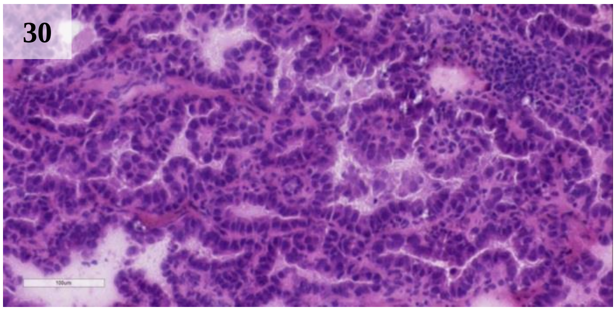
30

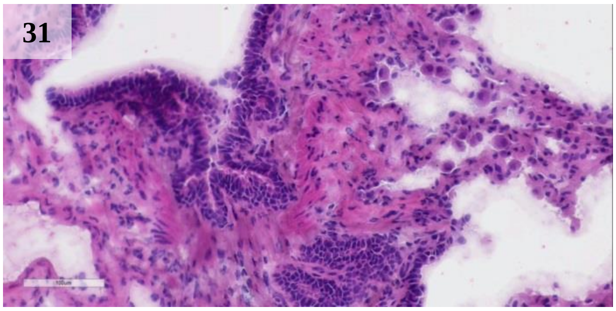
31

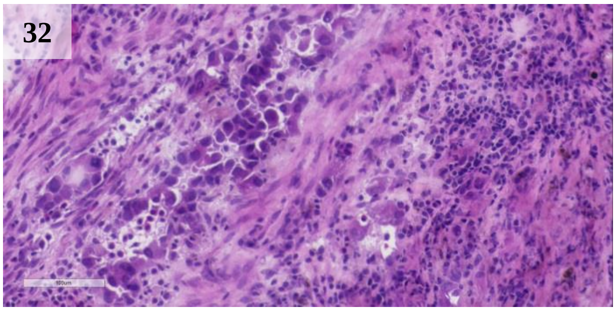
32

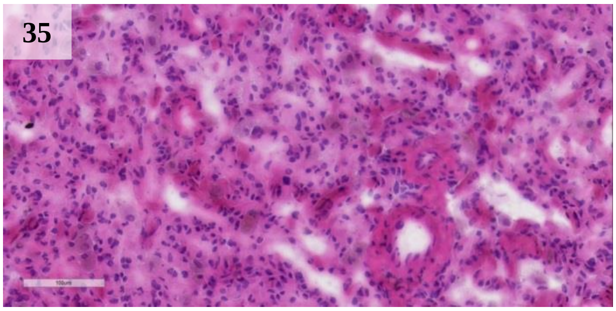
35

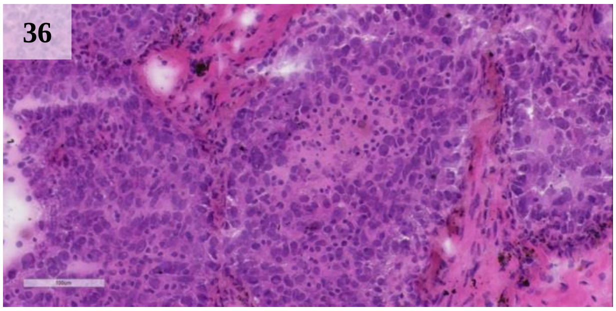
36

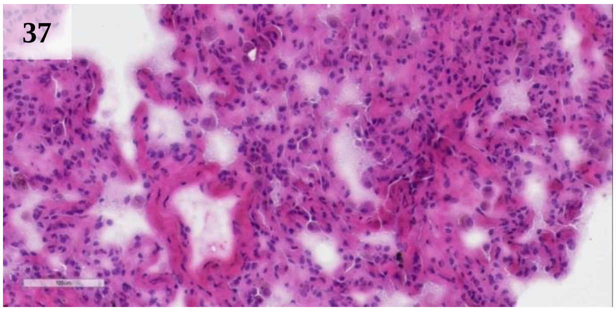
37

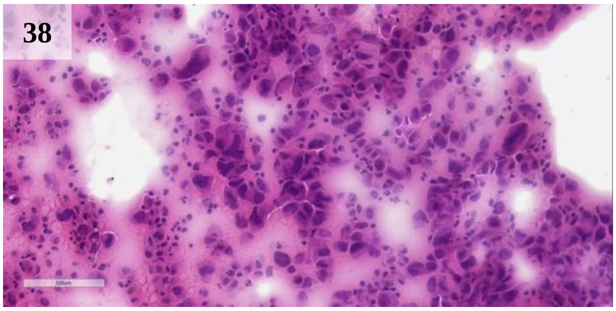
38

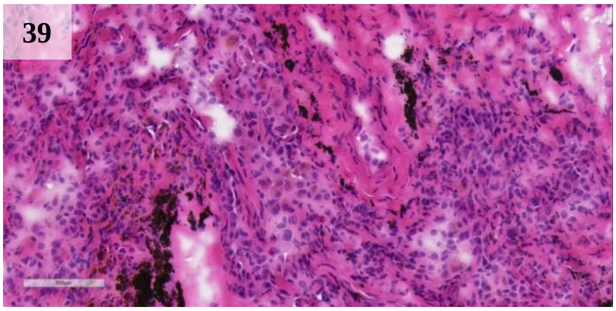
39

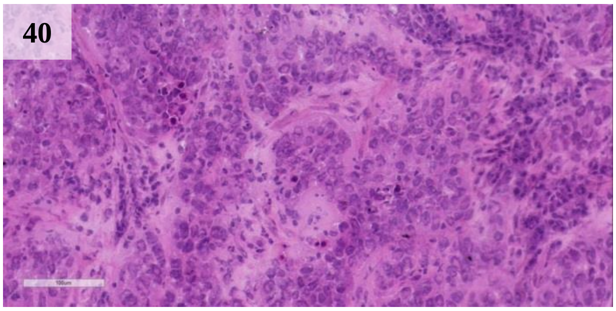
40

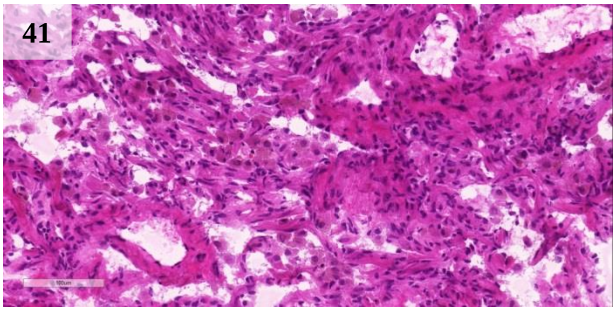
41

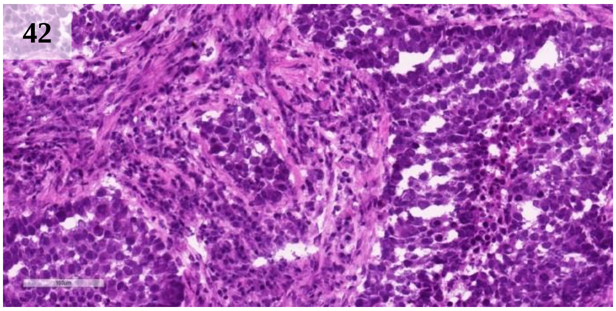
42

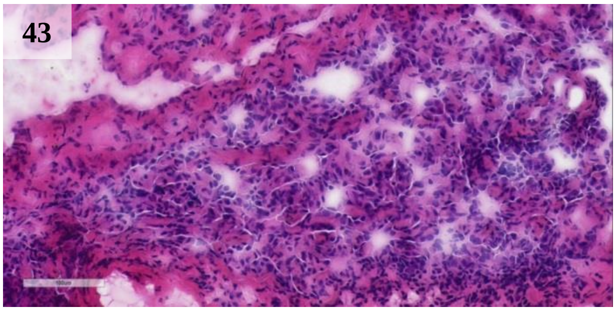
43

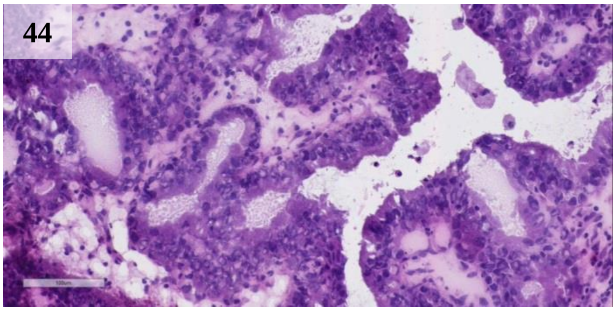
44

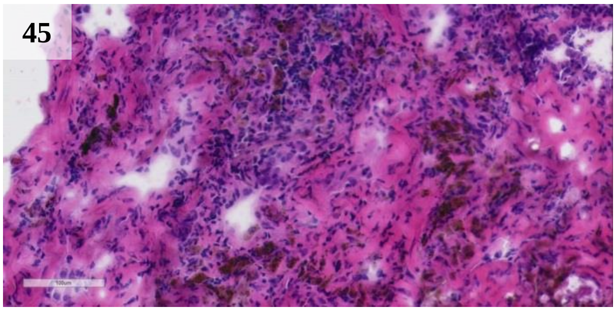
45

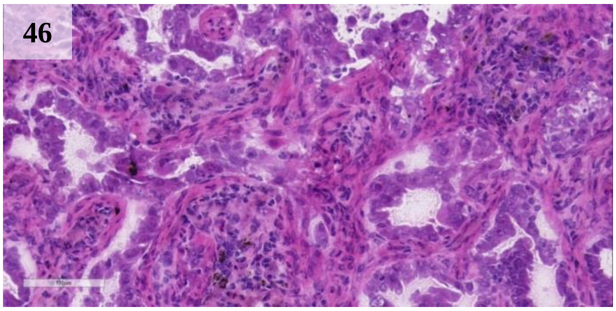
46

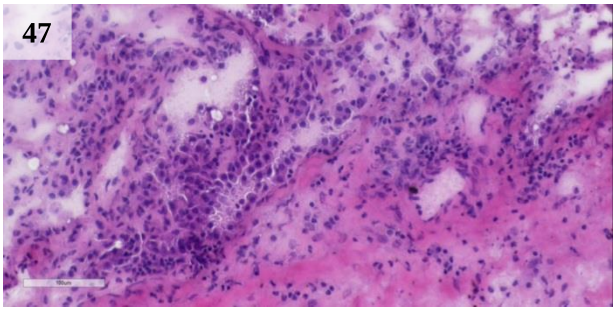
47

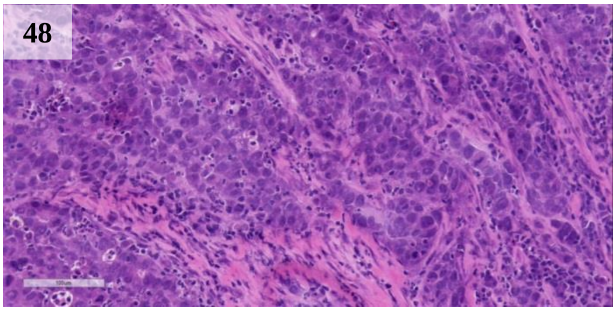
48

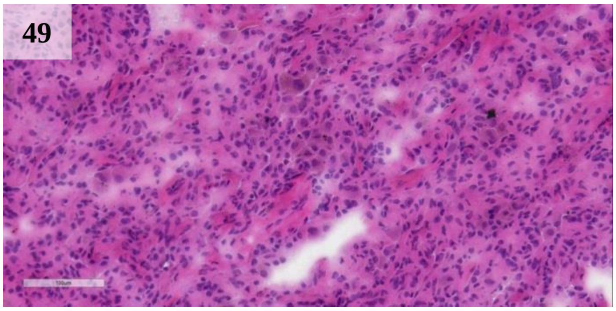
49

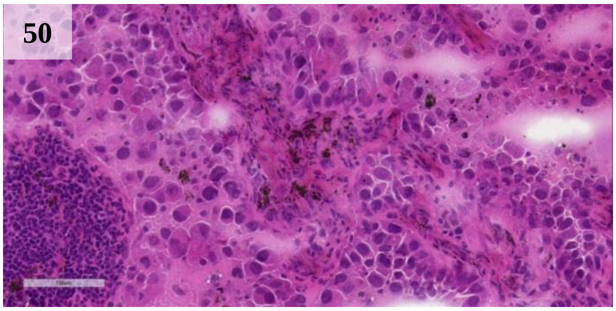
50
