# Supplementary material for: Proteomic analysis enables distinction of early‐ versus advanced‐stage lung adenocarcinomas
Source: Clin Transl Med. 2020 Jun 14;10(2):e106. doi: 10.1002/ctm2.106 (PMC7403673; doi:10.1002/ctm2.106)
Supplement: Supplementary file 4 — Supporting Information [file CTM2-10-e106-s005.pdf]

Supplementary File F2.

Quality control of LC-MS runs

Retention times and ΔMS

Four peptides were selected because of their high rate of observation in LC-MS analyses performed in our laboratory with tissue samples (**Figure F2-S1**). We reported the median of the retention times for the three replicated measured by sample. As we expected they were identified in all the samples analysed. The CV for all of them across all LC-MS/MS runs was lowers than 5% (**Figure F2-S1**). As an example, we showed in **Figure F2-S1** the results for four samples (one by condition studied) measured by triplicate. The CV of the retention time intra and inter conditions –sample was also always lower than 5% (**Figure F2-S1**). In addition, the selected chromatogram ranges for the peptides (base peak plot type), the MS1 and MS2 (most fragments assigned to their sequences) and the retention times distribution of the peptides in all samples measured (**Figure F1-S2**). The MS error distribution for peptides groups showed a median of around -1 ppm, and the distribution of all peptide groups by RT in the performed gradient is shown below in **Figure F2-S3**.

Figure F2-S1.

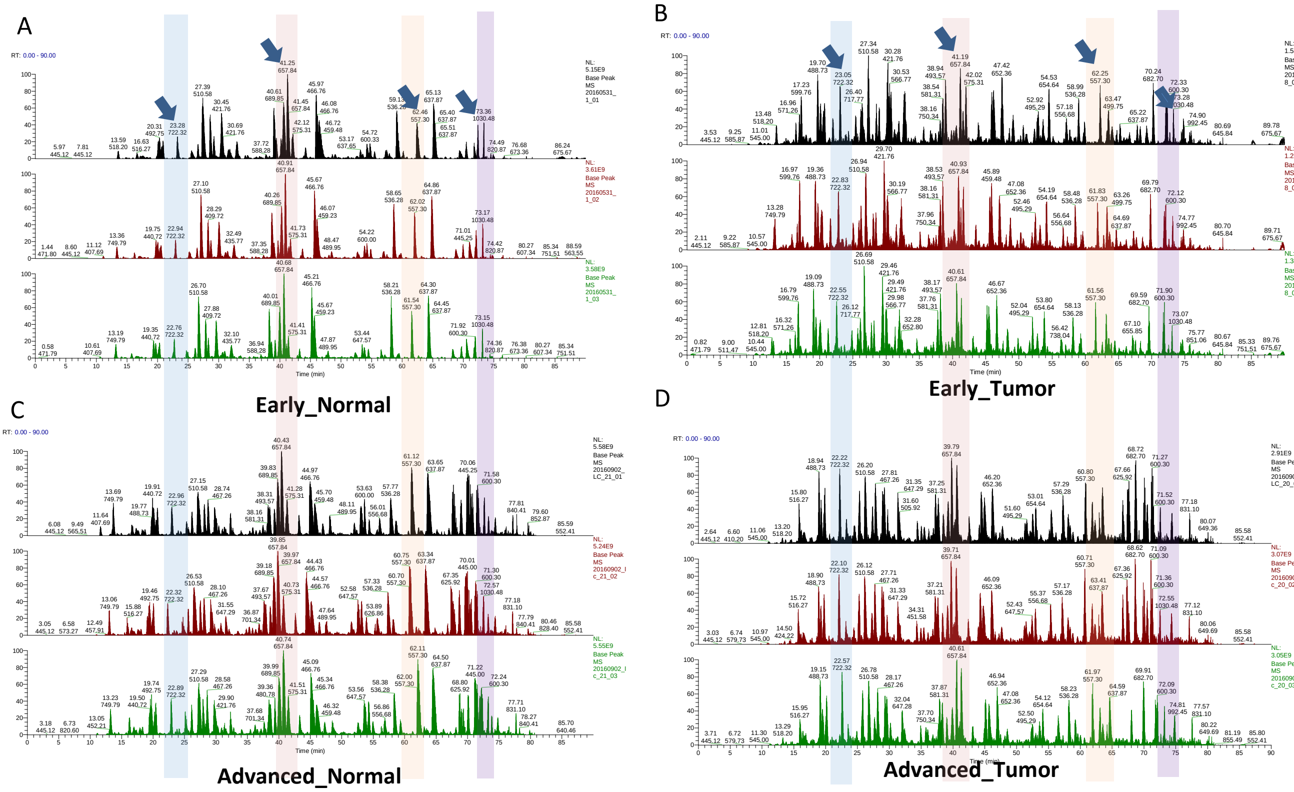

Sequence	Theoretical m/z	z	# samples identified	Delta ppm	Median RT	CV (RT) %
YICENQDSISSK	722.3246	2	ALL	0.09806318	23.229193	3.912628
VNVDEVGGEALGR	657.836	2	ALL	0.29647219	41.190885	3.458089
VLGAFSDGLAHLDNLK	557.3018	3	ALL	0.00940195	62.152602	1.975956
FFESFGDLSTPDAVMGNPK	1029.9775	2	ALL	0.51466945	73.208451	1.575471

Sequence	RT (early_normal)				RT (early_tumor)				RT (advanced_normal)				RT (advanced_tumor)				
	R1	R2	R3	CV (%)	R1	R2	R3	CV (%)	R1	R2	R3	CV (%)	R1	R2	R3	CV (%)	CV_Total
YICENQDSISSK	23.28	22.94	22.76	1.15	23.05	22.83	22.55	1.10	22.96	22.32	22.89	1.54	22.32	22.10	22.57	1.05	1.53
VNVDEVGGEALGR	41.25	40.91	40.68	0.70	41.19	40.93	40.61	0.71	40.43	39.85	40.74	1.12	39.79	39.71	40.61	1.24	1.29
VLGAFSDGLAHLDNLK	62.46	62.02	61.54	0.74	62.25	61.83	61.56	0.56	61.12	60.75	62.11	1.15	60.80	60.71	61.97	1.15	1.00
FFESFGDLSTPDAVMGNPK	73.36	73.13	73.15	0.17	73.28	73.17	73.07	0.14	72.70	72.57	72.32	0.27	72.58	72.55	72.31	0.20	0.52

Figure F2-S1. *MS chromatograms in base peak mode for 12 LC-MS runs representing the four conditions studied (four samples and three MS replicates each) A) Early Normal, B) Early Tumor, C) Advance Normal, D) Advance Tumor. Four peaks belonged to four endogenous peptides that were followed through all LC-MS runs. The peptide sequences and all CV calculated in the whole study are shown in the two tables below. Table (up): MS error, RT median, and CV for the four peptides measured in all samples studied. Table (down): Peptide sequences, RT, and CV for the peptides followed in the four samples measured by replicates. In gray the CV by sample. In yellow the total CV for the 12 LC-MS measured.*

Figure F2-S2.

I) **Peptide:** YICENQDSISSK

A

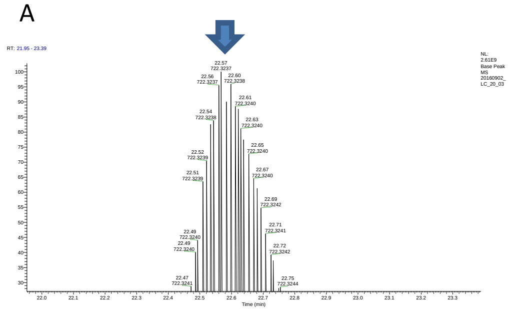

C

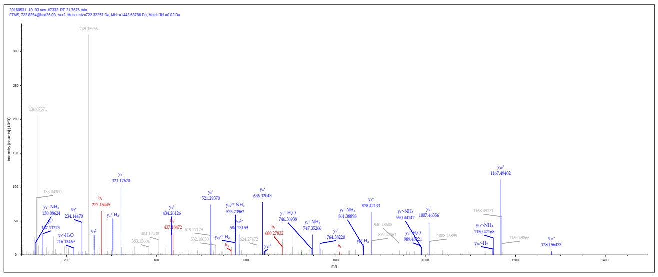

B

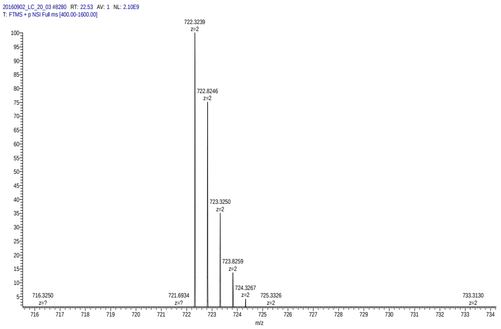

D

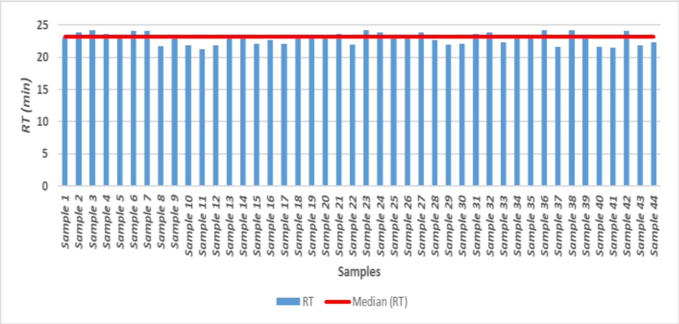

II) **Peptide:** VNVDEVGGEALGR

A

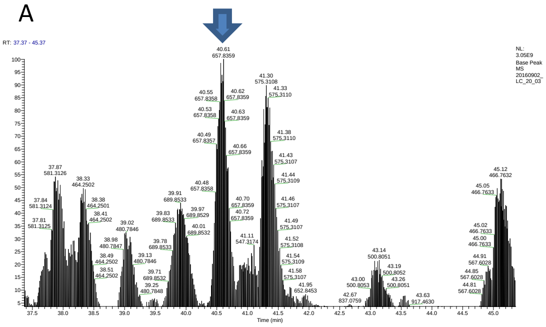

C

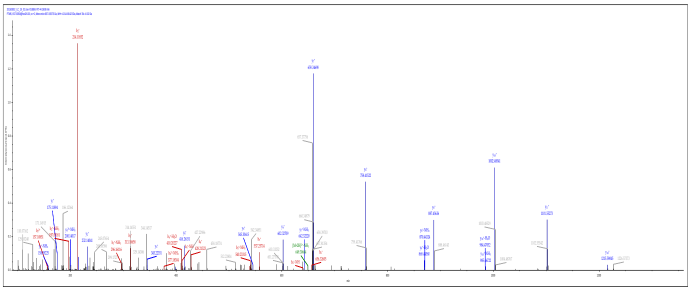

B

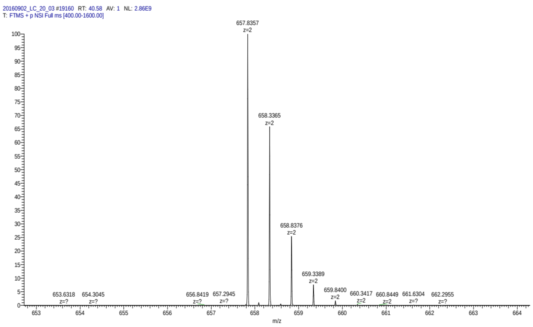

D

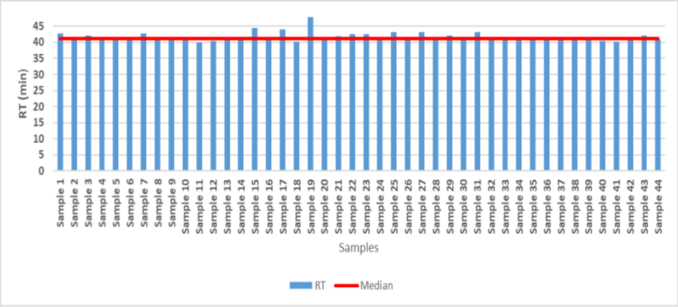

Figure F2-S2. *Examples of chromatograms, MS spectra, and RT distribution for the four peptides through all LC-MS runs. A) The Base peak for each peptide. B) MS1 spectra for each peptide. C) MS2 spectra (main fragments assigned to y and b series) for each peptide. D) Distribution of the median of the RT by samples for the peptides in all LC-MS runs (median by samples). A red line represents the median of the RT of the peptide in all runs.*

Figure F2-S2.

III) **Peptide:** VLGAFSDGLAHLDNLK

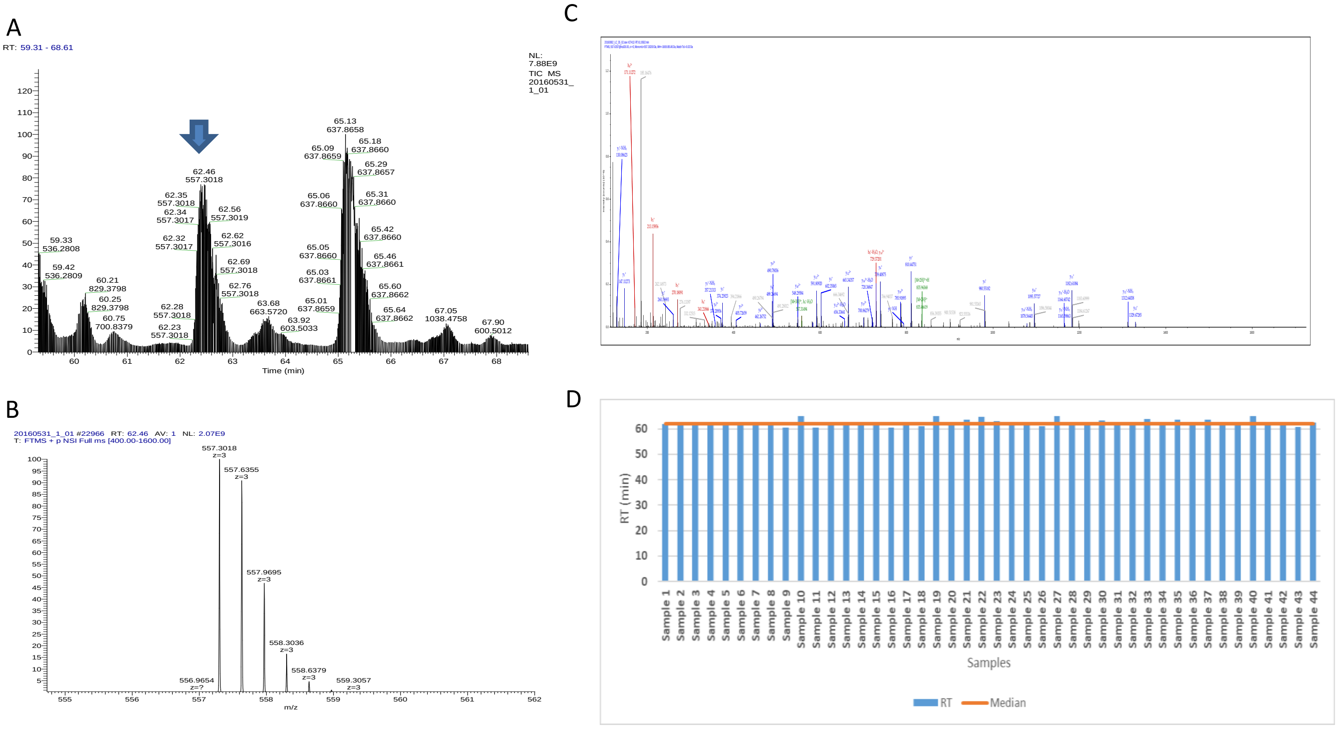

IV) **Peptide:** FFESFGDLSTPDAVMGNPK

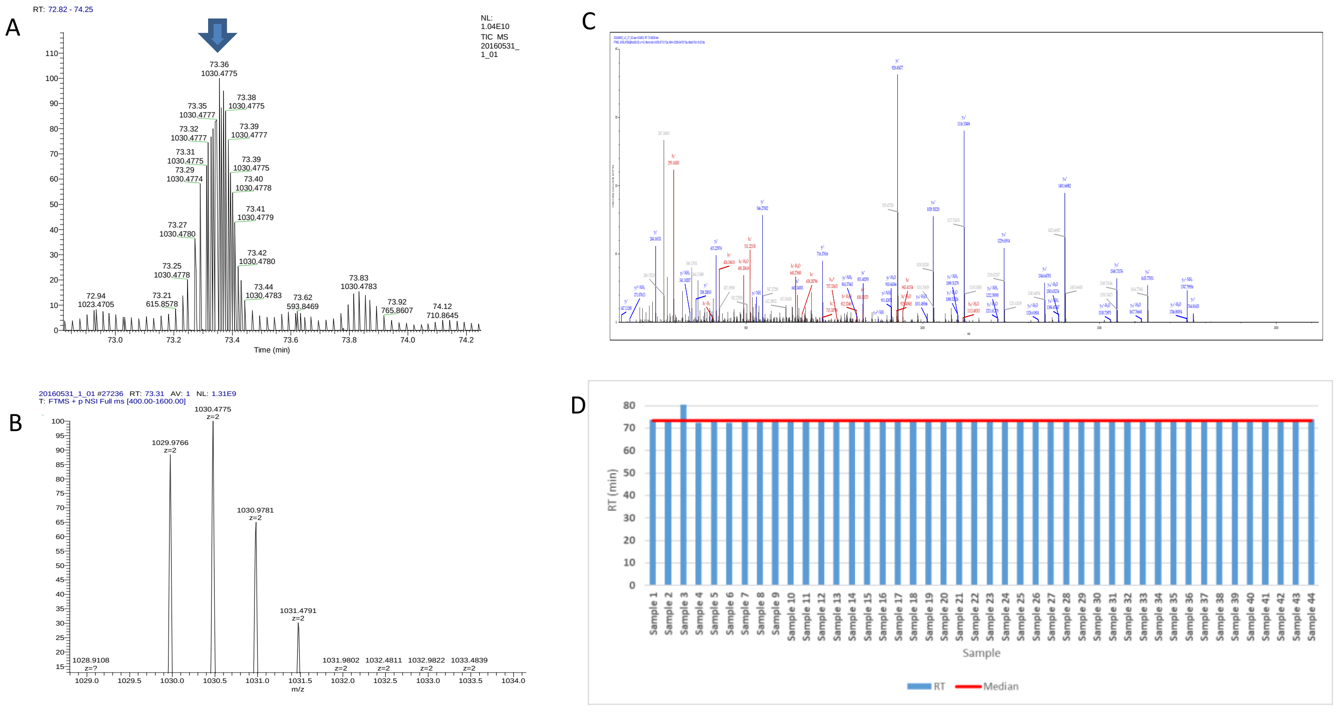

Figure F2-S2. *Examples of chromatograms, MS spectra, and RT distribution for the four peptides through all LC-MS runs. A) The Base peak for each peptide. B) MS1 spectra for each peptide. C) MS2 spectra (main fragments assigned to y and b series) for each peptide. D) Distribution of the median of the RT by samples for the peptides in all LC-MS runs (median by samples). A red line represents the median of the RT of the peptide in all runs.*

Figure F2-S3.

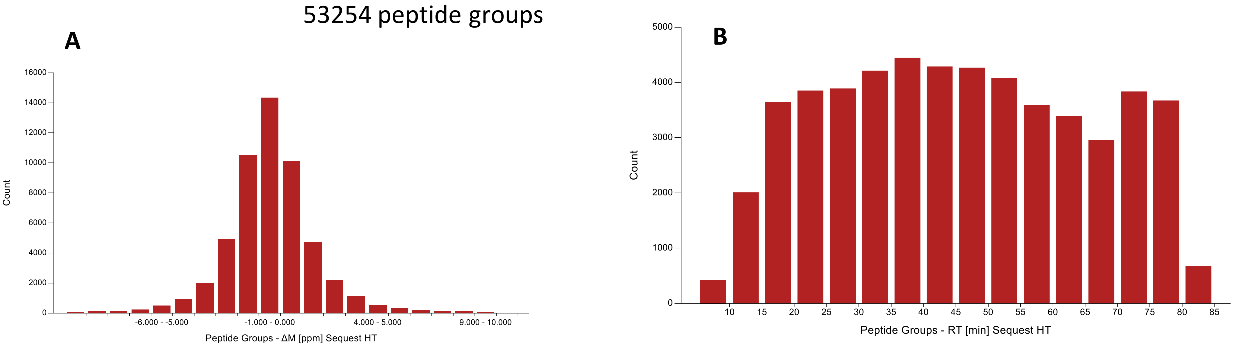

Figure F2-S3. *Mass error distribution for peptide groups (A) and distribution of all peptide groups by RT in the performed gradient (B).*
